# A proposed integrated approach for the preclinical evaluation of phage therapy in *Pseudomonas* infections

**DOI:** 10.1038/srep28115

**Published:** 2016-06-15

**Authors:** Katarzyna Danis-Wlodarczyk, Dieter Vandenheuvel, Ho Bin Jang, Yves Briers, Tomasz Olszak, Michal Arabski, Slawomir Wasik, Marcin Drabik, Gerard Higgins, Jean Tyrrell, Brian J. Harvey, Jean-Paul Noben, Rob Lavigne, Zuzanna Drulis-Kawa

**Affiliations:** 1 Department of Pathogen Biology and Immunology, Institute of Genetics and Microbiology, University of Wroclaw, Wroclaw, Poland; 2 Laboratory of Gene Technology, KULeuven, Leuven, Belgium; 3 Laboratory of Applied Biotechnology, Department of Applied Biosciences, Ghent University, Ghent, Belgium; 4 Department of Microbiology, Institute of Biology, The Jan Kochanowski University in Kielce, Kielce, Poland; 5 Department of Molecular Physics, Institute of Physics, The Jan Kochanowski University in Kielce, Kielce, Poland; 6 Department of Astrophysics, Institute of Physics, The Jan Kochanowski University in Kielce, Kielce, Poland; 7 National Children Research Centre, Dublin, Ireland; 8 Department of Molecular Medicine, Royal College of Surgeons in Ireland, Education and Research Centre, Beaumont Hospital, Dublin, Ireland; 9 Biomedical Research Institute and Transnationale Universiteit Limburg, School of Life Sciences, Hasselt University, Diepenbeek, Belgium

## Abstract

Bacteriophage therapy is currently resurging as a potential complement/alternative to antibiotic treatment. However, preclinical evaluation lacks streamlined approaches. We here focus on preclinical approaches which have been implemented to assess bacteriophage efficacy against *Pseudomonas* biofilms and infections. Laser interferometry and profilometry were applied to measure biofilm matrix permeability and surface geometry changes, respectively. These biophysical approaches were combined with an advanced Airway Surface Liquid infection model, which mimics *in vitro* the normal and CF lung environments, and an *in vivo Galleria* larvae model. These assays have been implemented to analyze KTN4 (279,593 bp dsDNA genome), a type-IV pili dependent, giant phage resembling phiKZ. Upon contact, KTN4 immediately disrupts the *P. aeruginosa* PAO1 biofilm and reduces pyocyanin and siderophore production. The gentamicin exclusion assay on NuLi-1 and CuFi-1 cell lines revealed the decrease of extracellular bacterial load between 4 and 7 logs and successfully prevents wild-type *Pseudomonas* internalization into CF epithelial cells. These properties and the significant rescue of *Galleria* larvae indicate that giant KTN4 phage is a suitable candidate for *in vivo* phage therapy evaluation for lung infection applications.

*Pseudomonas aeruginosa* is a metabolically versatile Gram-negative bacterium that can cause a wide range of opportunistic hospital-acquired infections. Individuals with open wounds, cancer, compromised immune systems and chronic pulmonary conditions, such as cystic fibrosis (CF) are particularly susceptible^1^,^2^,^3^. *P. aeruginosa* can undergo phenotypic and genotypic changes in response to the environmental signals during infection and lives as planktonic cells, colonies or biofilms^4^. The increasing frequency of multidrug-resistant strains is particularly concerning as treatment options are severely limited in the absence of effective antibiotics^5^,^6^. There is a general need to establish novel strategies for the development of new antibacterial treatments or effective prophylactics as well as new diagnostic tools to identify CF pathogen virulence determinants. One of the possibilities is bacteriophages, the natural parasites of bacteria. Phage cocktails have been applied as alternative or as supportive treatments simultaneously with antibiotics for *P. aeruginosa* eradication causing various infection such as purulent wounds, septicemia, urinary tract or lung infections^7^,^8^,^9^. A number of studies have presented phages with a potent bactericidal effect against CF isolates, reducing the lung bacterial burden in *in vitro* and *in vivo* models^10^,^11^,^12^,^13^,^14^. The capacity of phages to eradicate bacterial biofilms is an important aspect towards their successful implementation in *in vivo* treatments. To gain access to the bacterial cell, phages have developed specific strategies to overcome the biofilm. A major evolutionary development in this regard is the production of highly specific enzymes like polysaccharide depolymerases or alginate lyases. These enzymes allow the phages to invade the bacterial cells entrapped in the polysaccharide backbone by degradation of biofilm structure. Moreover, the bacteria released from exopolysaccharide matrix, become accessible for antimicrobials or host immune system components^15^,^16^,^17^,^18^,^19^. The phiKZ-like phages and their gene products may play an important role in control of pathogenic pseudomonads^20^,^21^. Some of these phages have already been incorporated into traditional phage therapy cocktails and continue to be examined for novel therapeutic applications^22^,^23^,^24^. *Phikzvirus* constitute a genus of ‘jumbo’ myoviruses, lytic against a variety of *Pseudomonas* species^25^,^26^. To date, over twenty phiKZ-related phages have been found in diverse geographic locations from soil, water, or have been recently characterized from historical therapeutic phage preparations^26^. PhiKZ-related phages have a large icosahedral head (~122 nm in diameter) and a long (~190 nm) contractile tail surrounded by fibers. Inside the capsid a large cylindrical “inner body” (15–20 MDa, consisting of at least six different proteins), holds the genomic DNA^27^,^28^. This long conserved head component spanning the whole giant capsid wall-to-wall was observed in phiKZ-related phages, e.g. 201 φ2–1, φPA3, EL, OBP^26^. Their genomes are very large (between 211 and 317 kb of non-redundant sequence) and compose of circularly permuted, terminally redundant linear double-stranded and A+T-rich (>52%) DNAs^29^.

This work describes the genome organization and biology of a novel phiKZ isolate KTN4, and its antibacterial potential is examined using novel biofilm assays (interferometry and profilometry) as well as a novel Airway Surface Liquid model on nonCF and CF epithelial cells lines, in an effort to mimic *in vivo* conditions of the respiratory tract as closely as possible. Indeed, the ASL covering the lung surface consists of a mucus and a periciliary liquid layer. The former traps and removes inhaled pathogens, while the latter keeps the mucus at an optimal distance from the underlying epithelia to maximize ciliary mobility, provides a low viscosity solution and acts as a lubricant layer for mucus transport. Together, they play a critical role in effective mucociliary clearance of the airway^30^,^31^. Due to mutations in the cystic fibrosis transmembrane conductance regulator (CFTR) in CF airways, ion channel abnormalities are observed and the innate and adaptive immune responses are impaired. Moreover, the decreased ASL volume or altered mucus impede phagocyte function, making bacterial elimination defective, inflammatory response abnormal, and mucociliary clearance reduced. CF airways have a sticky mucus providing the perfect milieu, microaerophilic to anaerobic environment, enabling the colonization and propagation of *P. aeruginosa*^32^.

## Results and Discussion

### Isolation and morphology

Lytic phage KTN4 was isolated from sewage samples collected from irrigated fields located in Wroclaw, Poland. After purification phage titres were 10^10^–10^11^ pfu/ml and caused ~1.7 mm wide clear plaques with halo zone on 0.6% soft agar. The KTN4 morphology was examined by transmission electron microscopy (TEM) and classified to the *Phikzlikevirus*, order *Caudovirales*, family *Myoviridae* (Figure S1)^25^. The isolate was formally named vB_PaeM_KTN4 (KTN4). The isometric head size can be estimated at 130 nm between opposite apices, the tail and base plate is 168 nm long.

### Molecular analysis and taxonomic context

**Figure 1:**
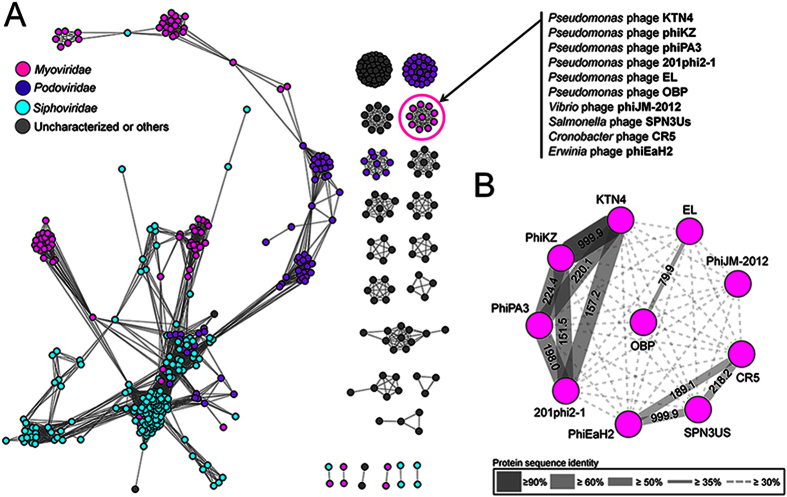
Protein-sharing network for KTN4. (**A**) A network representation was produced using the edge-weighted spring embedded layout of Cytoscape version 3.1.1. Nodes indicate phage genomes and edges between two nodes indicate their statistically weighted pairwise similarities with phage-phage similarity scores of ≥1. There are 495 nodes and 6,948 edges in this network. (**B**) An enlarged view of the circle in Panel B. Values are the similarity scores estimated with the hypergeometric equation shown in Materials and methods. Edge thickness is proportional to protein sequence identity, which is represented in the legend box.

Genome sequence analysis revealed KTN4 as a giant phage of 279,593 bp, highly similar (>99% genome-wide DNA homology, conserved regulatory elements and genome organisation) to *Pseudomonas* phage phiKZ. A detailed genome and proteome analysis is provided within a dedicated supplementary section, showing minor differences to phiKZ (Suppl. 1, Table S1, Figures S2–S4). An experimental structural proteome analysis (ESI-MS/MS) allowed the identification of 111 virion-associated gene products (out of 368 predicted open reading frames in total). To place this phage into its broader taxonomic context, a protein sharing network, comprising 495 *Caudovirales* and unclassified phages with 6,948 relationships (edges) between them, was generated (Fig. 1). As expected, phage KTN4 was placed in a single component with five well-known *Pseudomonas* phiKZ-related phages including phiKZ, phiPA3, 201phi2-1, EL and OBP^33^, as well as four other phages phiJM-2012, SPN3US, CR5, and phiEaH2, which was separated from other components.

### One-step growth and stability tests

One-step growth experiments indicated a latent period of 40 min and a burst size of about 6–8 phage particles per infected bacterial cell. The stability test revealed that KTN4 is relatively stable in a broad range of temperature and pH. No reduction of pfu/ml was observed over a period of 60 min at a temperature of 40–70 °C, while 1 hour incubation at 80 °C and 15 min incubation at 90 °C decreased the titer with 4 and 6 log, respectively. After 1 hour incubation at room temperature at pH between 6 and 12 over 90% phages remained infective. At a pH of 3–4 around 10% of particles showed the lytic activity, while pH 2 reduced the titer to less than 1%. No significant change in KTN4 titer was observed after 50% chloroform treatment for 1 h incubation at room temperature and at 4 °C.

### Determination of phage receptor and host range

In the study based on PAO1 mutants it was observed that KTN4 requires the presence of IV type pili on the surface of the host cell as its receptor (Table 1). The lytic activity of KTN4 was examined on two independent *P. aeruginosa* panels. In the previous paper, 43 clinical *P. aeruginosa* strains from COST international reference panel were used^14^. The KTN4 phage was able to infect 32.6% isolates, compared to representatives of the N4-like group (LUZ7 41.9%), the phiKMV-like group (LUZ19 44.2%), the PB1-like group (LBL3 39.5%, KT28 27.9%, KTN6 41.9%), the phiKZ-like group (phiKZ 46.5%) and a novel jumbo phage PA5oct 23.9%. In presented study, phage KTN4 exhibited a broad spectrum of activity against 58 clinical strains from Military Hospital Neder-Over-Heembeek, Brussels, Belgium^34^. KTN4 was able to infect 46.6% strains, whereas representatives of the N4-like group (LUZ7 34.5%, LIT1 12.1%), LUZ24-like group (LUZ24 22.4%), phiKMV-like group (LUZ19 39.7%, LKD16 31.0%, LKA1 3.5%, KMV 32.7%), and PB1-like group (LBL3 46.6%, LMA2 24.1%, LSL4 17.2%, KT28 58.6%, KTN6 67.2%) mostly show a narrower spectrum (Table S2).

An interesting observation here is the differences in host range between KTN4 and phiKZ, despite the strong homology between both phages. Indeed, tail sheath, tube, fiber, tip (gp39/181/40//182/166) and baseplate associated proteins (gp131-135) are all >98% identical to phiKZ. However, it was previously shown that only minor point mutations can result in host range differences^35^. A noticeable difference within the genome is associated to the presence of predicted HNH-endonucleases. It could be theoretically possible that their presence in DNA metabolism/structural protein coding genes could impact host range.

### Phage influence on biofilm characteristics covering Nephrophane membrane

**Figure 2:**
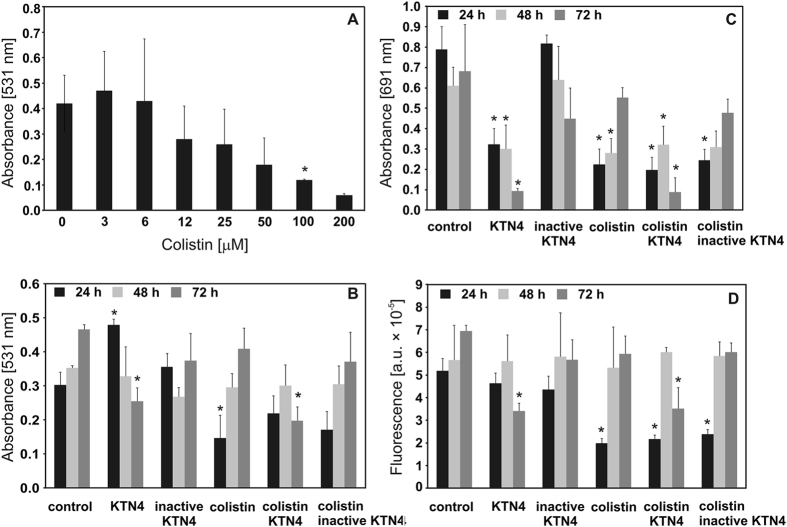
The antibacterial effect of colistin against 24 h PAO1 biofilm formed on Nephrophane membrane (**A**); the anti-biofilm effect of KTN4 phage/colistin treatment on 24, 48 and 72 h PAO1 biofilm formed on Nephrophane membrane: the biomass evaluation by CV staining (**B**); the level of pyocyanin in growth medium (**C**); the fluorescence of pyoverdin in growth medium (**D**). Untreated biofilm was used as control. The results are presented as the means ± SD. Statistical analysis was made by the ANOVA test (denoted *p*-values < 0.05).

The anti-biofilm activity of giant phage KTN4 was evaluated by spectrophotometry and a fluorescence assay, and compared with the efficacy of colistin, an anti-pseudomonal drug (Fig. 2). The activity of colistin was first adjusted on 24 h PAO1 biofilm growing in microtiter plates and the biofilm mass was evaluated by CV staining. Based on the above results, a concentration of 100 μM colistin was selected (Fig. 2A). Afterwards, experiments were performed on the PAO1 biofilm grown on a Nephrophane membrane for three time periods (24, 48 and 72 h). In the Fig. 2B, the CV staining of biofilm biomass eradication showed significant effect of active KTN4 and colistin combined with active phage against 72 h-old biofilm. The colistin alone significantly reduced the biomass of the biofilm formed for 24 h. In contrast, the CV assay showed an increase of biomass after intact phage particles treatment of 24 h biofilm. No biofilm mass eradication was noticed for inactivated phages regardless of biofilm age and the combination with colistin. In general, the CV staining did not reveal efficient eradication results both for phage and antibiotic. We suspect that phage propagation on the cells embedded in early biofilm leads to the release of exopolysaccharide degrading enzymes (of phage or bacterial origin), in turn resulting in a relative increase of negatively charged moieties in biofilm matrix. These moieties are detected during crystal violet staining as biofilm biomass increase. As discussed recently^17^, we have concluded that this phenomenon is a limitation of the CV assay. As a consequence, other methods have been applied to evaluate phage potency to affect biofilm forming bacteria. For this purpose, the analysis of pyocyanin and pyoverdin/pyochelin secretion to the medium by spectrophotometry and fluorometry was used, allowing detection of highly diffusible pigmented signaling molecules levels of quorum sensing and a siderophore^36^,^37^,^38^,^39^.

The analysis of pyocyanin concentration in growth medium showed that active phages, colistin and active phage/colistin treatment significantly decreased the level of this compound for the tested biofilms (24, 48 or 72 h) compared to UV-inactivated phages (Fig. 2C). It turned out that infective form of giant phage significantly reduced the concentration of pyocyanin, whether combined with colistin or alone. The level of pyoverdin determined by the fluorescence was significantly lowered after colistin application on 24 h biofilm and in combination with both phage preparations on PAO1 biofilm formed for 72 h (Fig. 2D). The reduction of siderophore concentration was also observed when 72 h biofilms were exposed to active KTN4. The combined treatment consisting of phage and antibiotic did not show a synergistic effect in either biomass or dyes determination assays. This observation is consistent with previous observations for two *Pseudomonas* PB1-like phages^17^. Moreover, the positive correlation between bacterial cells growth, biofilm formation, pyocyanin and siderophore levels in supernatants has been found, indicating that the reduced levels of *Pseudomonas*-specific compounds are related to the phage’s activity. Statistically significant (p < 0.005) inhibition observed for pyocyanin and pyoverdin/pyochelin production was elicited by phages (lysis of cells in biofilm). The quantities of the most important pigmented signaling molecules secreted by the pathogen decreased significantly, which proves the potency of these phages to be applied in *Pseudomonas* biofilm treatment.

**Figure 3:**
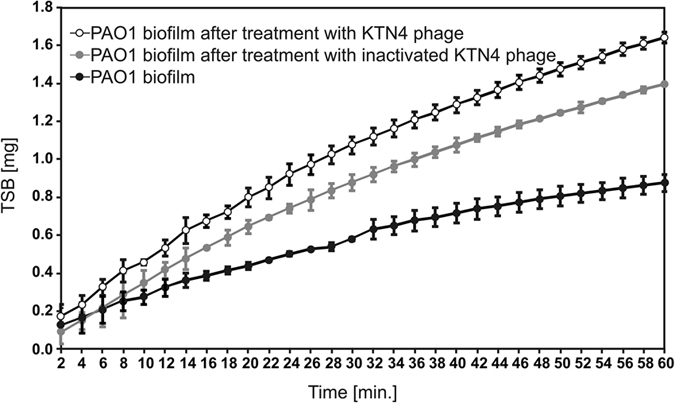
Laser interferometry analysis of TSB medium diffusion through PAO1 biofilm treated with phages. Untreated biofilm was used as control. The results are presented as the means ± SD from three independent experiments.

The phage application effect on biofilm disruption was analyzed by laser interferometry^17^,^40^, a biophysical technique which measures quantitative changes in biofilm matrix permeability for low molecular compounds (Fig. 3). This experiment was not performed for colistin, since no activity of the drug for three-days biofilm could be measured. The diffusion of TSB medium though biofilm was evaluated, which indirectly indicated its structure degradation. The hydrophilic Nephrophane membrane overgrown by 72 h biofilm was chosen for the interferometry analysis. At this moment, the PAO1 biofilm fully covers the Nephrophane membrane, necessary as a scaffold used in the laser interferometry method (Fig. 3). In addition, this 72 h time point allows comparison to other approaches (Fig. 2). ASM medium containing the components of cystic fibrosis patient sputum, including amino acids, mucin and free DNA was also attempted for biofilm culturing, as well. Unfortunately, the level of membrane coverage by PAO1 biofilm formed in ASM medium was too low (18–26%) and biophysical investigation by interferometry analysis proved problematic.

The PAO1 biofilm was treated with active and UV-inactivated KNT4 phage for 4 h. The diffusion rate of medium transported though the biofilm-covered membrane (0.86 × 10^−3^ mg/h) was significantly higher than for intact biofilm (p < 0.001) after active and inactivated phage treatment, reaching 1.65 × 10^−3^ mg/h and 1.39 × 10^−3^ mg/h, respectively. The increase of diffusion rate through the overgrown membrane after phage application indicated the degradation of the biofilm structure associated with the disintegration of matrix elements. It should be emphasized that the increase of the diffusion was also obtained after the application of inactivated particles, suggesting that tested phages may display an exopolysaccharide depolymerase, responsible for phage particle spread within the biofilm matrix. However, this could not be supported experimentally.

**Figure 4:**
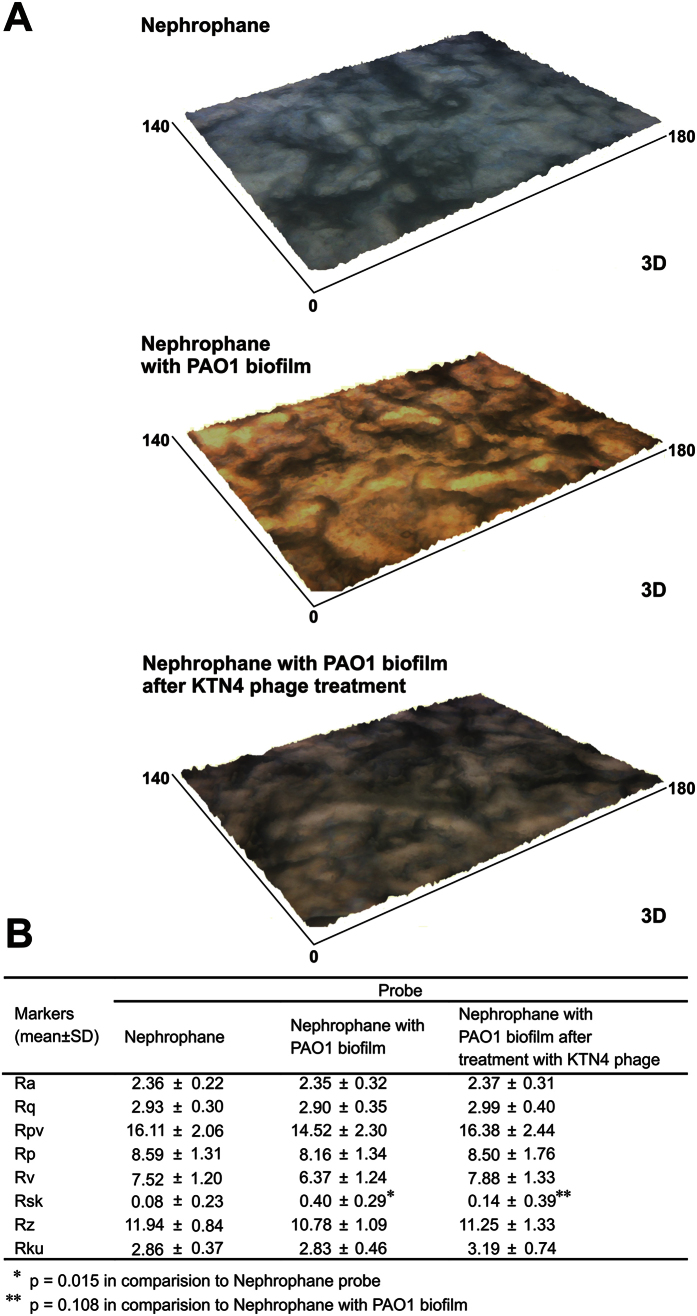
The 3D surface optical profile analysis of Nephrophane membrane, PAO1 biofilm and PAO1 biofilm after KTN4 phage bacteriophage degradation measured by ZET 20 instrument (**A**). The Nephrophane roughness (R) parameters measured by ZET 20 (**B**). The results are presented as the means ± SD. Statistical analysis was made using an ANOVA test (denoted *p*-values < 0.05).

The effect of KTN4 phage treatment on biofilm disruption was also analyzed by ZETA-20 non-contact optical profiler, a novel 3D measurement technique. Two of the system imaging modes (Z-Dot^TM^ and Nomarski (ZIC)) were used to determine the 3D profile of tested surface with the reflection on natural colors (Fig. 4). The yellow color of PAO1 biofilm (Fig. 4B) formed on the grey Nephrophane membrane was associated with the production of siderophores by bacterial cells within a mature biofilm. After the incubation with KTN4 phage, the color of degraded biofilm was similar to native membrane. It indicated that phage was able to inhibit the production of siderophore by PAO1 cells. Moreover, several parameters of surface roughness was also measured what allowed to analyze the physical changes in the biofilm geometry. The surface structure, described by Rsk parameter, has changed after incubation with phage preparation. The biofilm surface was smoother after phage treatment observed as the skewness parameter (Rsk) decreased from 0.40 ± 0.28 before treatment to 0.14 ± 0.39 post phage treatment (*p* = 0.108).

Considering the phage/colistin eradication efficacy of PAO1 biofilm, a rapid action of colistin compared to phages was observed. This could be associated with differences of diffusion kinetics between both tested antibacterial agents. The laser interferometry analysis of colistin diffusion (from initial concentration 1.2 mg/ml) through PAO1 biofilm formed for 72 h at 37 °C showed that only 12.17 μg/ml (about 1%) of colistin was transported after 40 min (data not shown). This indicates that the concentration of colistin in the old PAO1 biofilm was at a sub-inhibitory level. In contrast, the concentration of antibiotic likely reaches a bactericidal level in the early biofilm. The possible answer for the lack of synergy between phage and antibiotic can be explained by the mode of action and receptor targeted by each antibacterial agent. Colistin causes the cell death by destabilizing cell membrane, which limits phage propagation. In addition, KTN4 phage recognises IV-type pili and therefore neither increases nor inhibits colistin activity. In Fig. 2, both antibacterials work independently and the bactericidal activity was cumulated, yet not greater than the effect of each individual compound. Considering anti-biofilm assays in general, it seems that when comparing the CV assay to the novel approaches such as laser interferometry and profilometry (ZETA-20, Zeta Instruments Co., San Jose, USA), the latter turned out to be more efficient and precise techniques, providing important insights on the degradation and permeability feature of biofilm matrix, and surface geometry changes including several roughness (R) parameters and diffusible pigmented signaling molecules production by biofilm forming bacteria.

### Antibacterial efficacy of KTN4 phage analyzed in gentamicin exclusion assay on Airway Surface Liquid infection model

**Figure 5:**
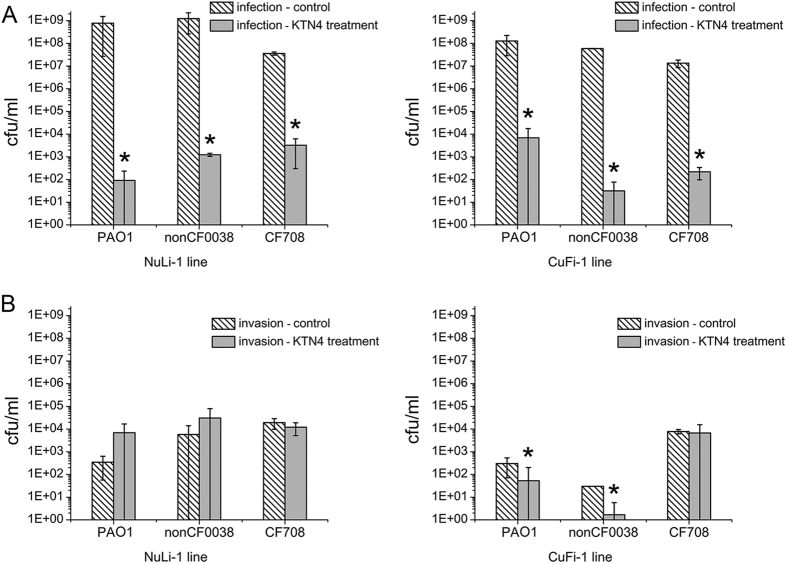
Phage KTN4 treatment of *P. aeruginosa* infected NuLi-1 and CuFi-1 epithelial cells. (**A**) Colony count of bacteria collected from apical wash; (**B**) colony count of bacteria internalized in epithelial cells. The results are presented as the means ± SD. Statistical analysis was made by the ANOVA test (denoted *p*-values < 0.05).

In our study, *in vitro* antibacterial activity of KTN4 phage was assessed in gentamicin exclusion assay on ASL, which is to our knowledge, the first report showing phage treatment efficacy in that infection model. For experiments, two cell lines were selected: 1) NuLi-1 derived from normal human bronchial epithelium and 2) CuFi-1 derived from CF patient bronchial epithelium with significantly thinner ASL^31^. Three *P. aeruginosa* strains were selected for these experiments: PAO1 reference strain (piliated, motile strain, effective biofilm former), a nonCF0038 isolate from burn wound (highly expressing type IV pili), both reflecting CF early colonizing isolates, which are non-mucoid with typically smooth LPS and more virulent. The third strain was CF708 from late infection phase, presenting slowly growing and less virulent small colony variants (SCVs) with low expression of type IV pili, and biofilm formation^18^. First, both epithelial cell lines were infected with selected strains for 3 h and colony count showed *P. aeruginosa* efficiently propagated in both ASLs (10^7^–10^9^ cfu/ml) (Fig. 5). Although PAO1 and nonCF0038 pathogens grew better on NuLi-1 cell line, reaching around 1 log higher compared to CuFi-1 ASL, no statistics significance has been found (*p* > 0.05). The small colony variant CF708 grew equally well in both types of mucus layer. In the second step of experiment, the KTN4 phage eradication ability of the extracellular bacterial load was evaluated. CFU counts of *P. aeruginosa* were significantly (*p* < 0.05) reduced for the normal NuLi-1 epithelia cells. A 7 log, 6 log and 4 log decrease was observed for PAO1, nonCF0038 and CF708, respectively (Fig. 5A). In the case of the CuFi-1 epithelia the phage treatment was also very effective giving 4 log, 6 log and 5 log reductions in colony count of PAO1, nonCF0038 and CF708 (*p* < 0.05), respectively (Fig. 5A). PAO1 was significantly more susceptible (*p* < 0.05) to phage treatment in NuLi-1 cells compared to CuFi-1 cells, contrary to the CF708 isolate. It was confirmed that the KTN4 phage could freely diffuse and gain access to the bacterial hosts in both ASL models, but the final result of the treatment was strongly dependent on the strain features or due to different ASL pH between NuLi-1 and CuFi-1 cells. A possible explanation of this phenomenon was observed by Worlitzsch’s studies^32^ where *P. aeruginosa* was not interacting with the CF epithelium directly, but was rather found trapped in mucus plugs formed in the airways. Thus, the phage receptors could be masked by mucus elements, which have an influence on phage adsorption to bacterial cell surface. Moreover, the CF strain is better adapted to CuFi-1 environment and may presumably express the type IV pili more efficiently, which are receptors for KTN4 phage.

In the next step of our experiment, the ability of *P. aeruginosa* strains to invade into epithelial cells was investigated (Fig. 5B). Although *P. aeruginosa* was generally thought to be an extracellular pathogen, a number of different groups have found that it can be internalized into a range of different cell types, including epithelial cells^3^,^41^. The results showed that CF and non-CF strains could indeed internalize into both cell lines with no statistically significant differences (*p* > 0.05) were observed for all pathogens. Nonetheless, the PAO1 and nonCF0038 were much less ingested by epithelium compared to the CF708 isolate, where only 4.50E-05% and 2.00E-04–5.00E-05% of infecting population were able to internalize into NuLi-1 and CuFi-1 cells, respectively, in contrast to 0.05% and 0.06% for CF708 isolate. Clearly the internalization capability and adaptations of CF708 strain were more effective compared to the nonCF strains, consistent with previous observations^41^. This suggests that the *Pseudomonas* strains with high cytotoxicity are low invasive and vice versa, bacteria less virulent enter the epithelial cells to survive intracellularly without killing the host cell.

In the final step, the influence of KTN4 phage treatment on the number of invaded bacteria was evaluated (Fig. 5B). In the NuLi-1 cell line there were no significant changes (*p* > 0.05) in CFU counts for all *P. aeruginosa* invading strains after phage application. A possible explanation is that during the 3 hours of pretreatment, all bacterial cells were already internalized, thus the phage had no access to these host cells. In contrast, the phage application was significantly more effective (*p* < 0.05) for CuFi-1 internalization prevention by wild type *P. aeruginosa* strains, since CF lung cells due to lack of CFTR, show significantly less ingestion rate of LPS-smooth bacteria and significantly greater lung burdens post-infection than wild-type epithelium^3^. As previously mentioned^32^, wild strains not well adopted to the ‘sticky’ and dense CF environment are absent on epithelial surface but remain as macrocolonies within intraluminal material, slowing down efficient internalization. Simultaneously, phage application cause effective eradication of bacterial cells trapped in the mucus plugs. The CF708 isolate evolving in CF patient is able to internalize with the CuFi-1 epithelium in relatively short time after infection evading phage lytic activity.

### Antibacterial efficacy of KTN4 phage analyzed *in vivo* on wax moth larvae model

**Figure 6:**
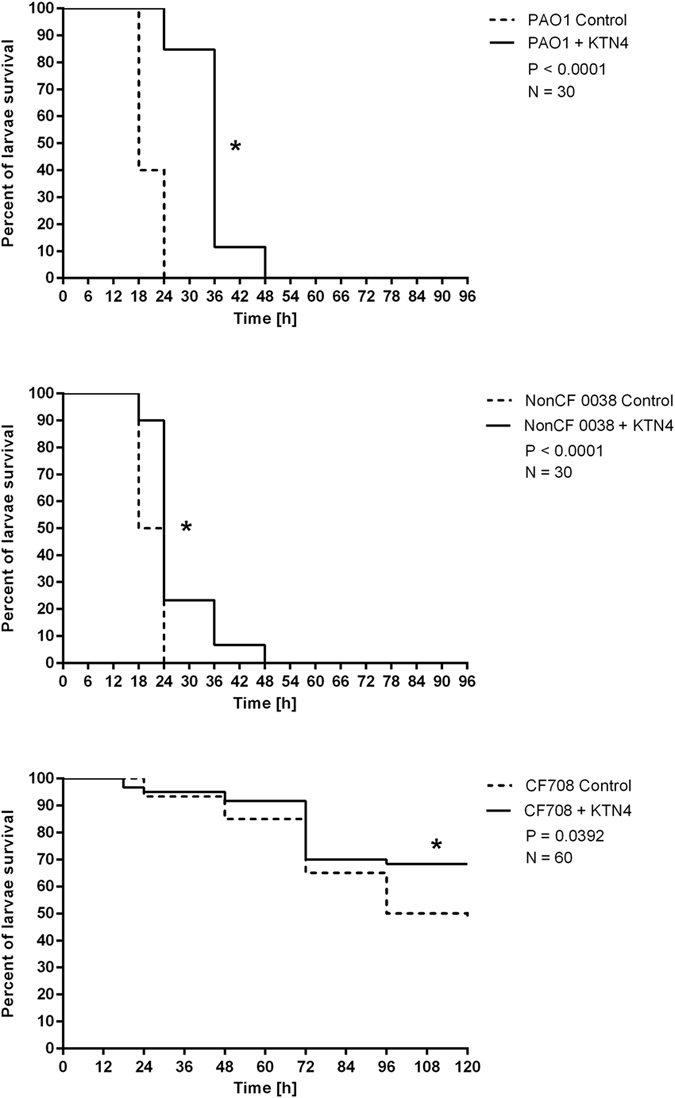
Antibacterial activity of KTN4 phage (MOI 100) in the treatment of infected *Galleria* larvae by *PA* strains. Positive control consisted of infected but untreated larvae and KTN4 control was larvae group receiving phage lysate only. Statistical analysis was calculated for pair wise comparisons between infected larvae and phage treated infected larvae using Mantel-Cox test (denoted *p*-values < 0.05).

The wax moth larvae model has been chosen for the *in vivo* assay because the *P. aeruginosa*, as a natural *Galleria* pathogen, is highly virulent in these insects when inoculated directly into the hemolymph^42^,^43^. The lethal dose causing fast infection progress was established as follows: 10 CFU for PAO1 and nonCF0038 strains and 10^6^ CFU of CF708 isolate per larvae and the treatment was carried out by the injection of phage lysate at multiplicity of infection (MOI) of 100 (Fig. 6). The negative controls (uninfected and receiving phage lysate larvae (N = 30 per condition) gave a 100% survival rate. The 10 CFU of PAO1 and nonCF0038 strains caused 100% caterpillar mortality (N = 30 per condition) after one day of infection. The small colony variant CF708 isolate even at very high inoculum (10^6^ CFU) was significantly less virulent than former ones (*p* < 0.0001) with the delay of killing 35% and 50% after three and four/five days of infection (N = 60), respectively.

The KTN4 phage application showed a significant impact on *Galleria* larval survival rate from lethal PAO1 infection, with 90% of alive caterpillars 36 h post injection (*p* < 0.0001). The protective activity of the phage against nonCF0038 propagation was much less efficient, saving only 20% of larvae at the same time (*p* < 0.0001). The antibacterial activity of applied phage against CF708 isolate was seen at the very end of the experiment (fourth/fifth day) with 68% survival rate of treated larvae (*p* > 0.05) in comparison to 50% of untreated control.

The results obtained in *G*. *mellonella* model generally correlate with data observed for NuLi-1 cells in ASL infection assay, where the bacterial count of PAO1 was reduced by KTN4 phage more efficiently than of nonCF0038 strain, although the later was described as a strong type IV pili former (the principal KTN4 receptor). The possible explanation of this effect could be the biochemical clonally variation of non-CF308 population examined by Fourier transform infrared spectroscopy (FTIR) analysis in the spectra window of carbohydrates and lipids, in our previous study^18^. This may influence the stronger variation of phage susceptible cells among treated non-CF308 population. Comparing presented *Galleria* experiment to our previous study performed on the same strains^18^, the protective efficacy (*p* < 0.0001) of KTN4 phage was similar to the activity of another giant phage PA5oct tested in Olszak *et al*. study, which rescued 90% of caterpillars after two days of PAO1 infection. The increased larval survival rate in the presence of PB1-like phage (100% at the same time) compared to the giants ones, could suggest the importance of phage size and phage generation time rate (KT28 < PA5oct and KTN4) in the therapeutic results^18^.

In the moth larvae model, an antibacterial potential of KTN4 phage was shown against *P. aeruginosa*. There are some arguments that the administration of bacteriolytic agent (antibiotic, phage) in the treatment of infection, especially caused by Gram-negative bacteria, may result in severe consequences as Systemic Inflammatory Response Syndrome due to the rapid release of lipopolysaccharide (LPS) during lysis of a big number of cells in a short period of time. Nonetheless, the acute infection phage therapy data, as well as for bactericidal antibiotics, have shown that removing the pathogen population has profound effect on reducing proinflammatory cytokines leading to successful treatment.

## Conclusions

Genome and proteome analysis, as well as a protein-sharing network indicates that KTN4 phage belongs to “jumbo” *Myoviridae* and it is closely related to phiKZ phage. This lytic virus has a broad spectrum of activity with prevalence to clinical isolates, especially from CF patients. Moreover, this phage has a very strong bactericidal effect (4–7 log reduction of colony count) against *P. aeruginosa* strains, as tested in a ASL model. To our knowledge this is the first study of phage application using this lung epithelia infection assay. The gentamicin exclusion assay on ASL *in vitro* model is flexible, generates reproducible data with well-controlled and standardized conditions, mimicing the normal and CF lung environments. Moreover, it provides a basis for understanding the host-pathogen interactions and is, as such, an important step towards experimental *in vivo* studies. Indeed, the *Galleria* larvae model provides a first confirmation of the *in vivo* potential of the antibacterial efficacy of KTN4 phage against clinical isolates, albeit in a strain dependent manner.

Since *Pseudomonas* common infections are usually associated with biofilm formation, the ability of KTN4 to disrupt the biofilm has been examined in detail. The phage demonstrated a strong anti-biofilm potential immediately after application. In contrast to the commonly used CV assay, novel biophysical techniques (interferometry and profilometry) have proven to be sensitive and reproducible techniques, providing information on changes in biofilm permeability and the 3D structure, during the biofilm structure degradation process.

Considering all these characteristics, KTN4 phage is a suitable and promising candidate for *in vivo* trials, for applications in treatment and prophylaxis in lung infections.

## Methods

### Isolation, propagation and purification of phages

The *Pseudomonas aeruginosa* PAO1 (ATCC 15692) strain was used as phage propagation host. Environmental water samples from irrigated fields in Wroclaw, Poland were centrifuged (15,000 g for 15 min) and the supernatant was filtered through a 0.22 μm Millex-GP filter (Merck Millipore, Germany) to remove bacterial debris. *Pseudomonas* phage KTN4 has been propagated as previously described^17^. Phage lysate was purified with CsCl-gradient ultracentrifugation as described by Ceyssens *et al*.^44^. The phage titre of the solution was assessed using the double-agar layer technique^45^. The virion morphology in transmission electron microscopy (TEM) was established according to the method described elsewhere^17^.

### DNA isolation and sequencing

Phage DNA was isolated according to the modified protocol for λ DNA isolation^46^ after CsCl gradient purification of phage particles (10^10^ pfu/ml). Whole genome sequencing was performed by use of the Illumina MiSeq platform available at the Nucleomics Core (VIB, Belgium). A 2*150 bp paired-end library (Nextera XT sample prep) was prepared and sequenced. The reads were assembled in a single contig with a 100–6000 fold coverage using CLC genomics Workbench *de novo* assembly algorithm (CLC bio, Qiagen Company). The genome of bacteriophage KTN4 was deposited at GenBank under accession number KU521356.

### *In silico* genome analysis

Potential ORFs were identified using the GeneMark S^47^, GeneMark.hmm^48^, OrfFinder^49^ and manually analyzed. Translated ORFs were compared to known proteins using BLASTP^50^, the HHpred server^51^ and HMMER^52^, providing further insight into the predicted function of proteins. Conserved protein domains were identified using the Pfam^53^, InterPro^54^ and PHYRE2^55^. Putative tRNA genes were searched for using the tRNAscan-SE program^56^. The intergenic regions were screened for regulatory elements using fuzznuc^57^ and manually evaluated. Putative factor-independent terminators were identified with ARNOLD software^58^.

### Protein family clustering and network construction and analyses

To represent the genetic relationships of KTN4 with other phages as a gene (protein)-sharing network, each predicted protein was clustered into protein families using the ACLAME database (version 0.4)^59^ with the database of “viruses” and an E-value < 0.001^60^. Additionally, for the phages that share significant gene contents with KTN4 but are absent in the ACLAME database, 2,592 protein sequences were retrieved from phiKZ (NC_004629), phiPA3 (HQ630627), 201phi2-1 (NC_010821), EL (NC_007623), OBP (NC_016571), phiJM-2012 (JQ340088), SPN3US (NC_027402), CR5 (NC_021531), and phiEaH2 (NC_019929). The proteins that could not be assigned into any ACLAME protein families were defined as the unclassified protein families (UPFs) as previously described^61^. We accepted the transitive nature of sequence families^62^, i.e., a sequence is added to a cluster if it shares a reciprocal best hit relationship with at least one of the sequences of the cluster. The degree of similarity between other phages was generated as the minus logarithmic score by multiplying hypergeometric similarity *P*-value by the total number of pairwise comparisons^60^. Afterwards, a protein-sharing network was built with the Cytoscape software platform (version 3.1.1; http://cytoscape.org/), using an edge-weighted spring embedded model. Topological properties of the network were estimated with the Network Analyzer 2.7 Cytoscape plug-in^63^.

### ESI-MS/MS analysis of structure-associated proteins

Phage proteins were extracted from a purified phage suspension (10^11^ pfu/ml) by a single methanol/chloroform extraction (1:1:0.75, v/v/v) (Acros Organics) and subsequently precipitated by addition of an equal volume of methanol (16,060 *g*, 6 min). The dried phage protein pellet was resuspended in SDS-PAGE loading buffer and boiled for 5 min before loading onto a 12% SDS-PAGE gel. Protein gels were stained afterwards with GelCode Blue Safe (Thermo Scientific). Further, the entire lane of a phage protein profile was prepared for ESI- MS/MS as previously described^64^,^65^.

### Burst size experiments and Sensitivity of phage particles to heat, chloroform and pH

A one-step growth curve was performed according to the method of Pajunen *et al*.^66^ with modifications. An equal volume of bacterial culture (at optical density at 600 nm of 0.4) was mixed with phage suspension (10^6^ pfu/ml) to obtain a multiplicity of infection of 0.01. Phages were allowed to adsorb for 8 min at 37 °C, after which the mixture was diluted to 10^−4^. Triplicate samples were taken during 1 h at 5 min intervals and titrated. The sensitivity of phage particles to heat, chloroform and pH were performed according to previously described methods^17^.

### Phage typing and phage receptor analysis

The lytic activity of KTN4 was examined on 58 clinical strains from Military Hospital Neder-Over-Heembeek, Brussels, Belgium collection^34^ for comparison to other *Pseudomonas* phages including: N4-like group (LUZ7, LIT1), LUZ24-like group (LUZ24), phiKMV-like group (LUZ19, LKD16, LKA1, KMV), PB1-like group (LBL3, LMA2, LSL4, KT28, KTN6) (Table S3). Moreover, the phage specificity to particular bacterial receptor was tested on PAO1 mutants deficient in biosynthesis of A-band and B-band O-antigen, flagella, IV type pili, or alginate production (Table 1). For all phage experiments 4–6 h old bacterial cultures were used, unless otherwise stated. To determine bacterial susceptibility to phage-mediated lysis, a drop of the phage suspension (10^8^ pfu/ml) was put on a bacterial lawn and incubated at 37 °C. The plates were checked after 4–6 h and again after 18 h for the presence of a lysis zone^67^.

### Phage influence on biofilm characteristics covering Nephrophane membrane

Nephrophane (VEB Filmfabrik, Wolfen, Germany) is a microporous, highly hydrophilic membrane made from cellulose acetate ([trio-acetate cel-(OCO-CH_3_)n]) of a spongy structure^40^,^68^. The analysis of biofilm degradation by intact and UV-inactivated phages was performed by microbiological methods as well as biophysical techniques. In the first step of the study, the antibacterial effect of colistin against PAO1 biofilm formed in TSB medium for 24 h at 37 °C was determined. This step was made in microtitre plates using the CV (0.004% crystal violet) assay. Afterwards, the Nephrophane membrane was covered by a PAO1 biofilm formed for either 24, 48 or 72 h at 37 °C in TSB medium. Next, the biofilm was treated for 4 hours with KTN4 phage (5 × 10^8^ pfu/ml) and/or colistin at a 100 μM concentration at 37 °C. After incubation, the biofilm was stained with CV (0.004%) for 15 min or was tested for pyocyanin and pyoverdin/pyochelin levels in the supernatants, as previously described^17^. Pyoverdin and pyochelin are the two major siderophores produced by *Pseudomonas aeruginosa* in iron-limited media. The lex for pyoverdine is between 380–400 nm (depending on pH) and lem is 447 nm, lex for pyochelin is 313 nm and lem is 430 nm^69^. We decided to use lex = 398 nm and lem = 460 nm as more specific for pyoverdin in our experiment, but we cannot exclude (partial) detection of the pyochelin portion in the detected signal of siderophores due to spectral overlap. The degradation of biofilm by phages is associated with increase of the permeability of its matrix for low molecular compounds. The quantitative measurements of cultivation medium (TSB) diffusion through biofilm structure after incubation with phages might indicate on disruption of its structure. This degrading activity of KTN4 phage on PAO1 biofilm was tested by laser interferometry method as presented elsewhere^17^,^40^. The level of Nephrophane membrane covered by biofilm was determined as 92.4%. The interferometry system consisted of two glass cuvettes, separated by the horizontally located Nephrophane membrane, covered with PAO1 biofilm formed for 72 h at 37 °C. The KTN4 phage treatment (5 × 10^8^ pfu/ml) was carried out for 4 h at 37 °C. All experiments were performed in triplicate.

PAO1 biofilm surface geometry, its physical parameters as well as true color of 3D optical profile were determined by ZETA-20 (Zeta Instruments Co., San Jose, USA). Two imaging modes, Z-Dot^TM^ Optical Profiler and Nomarski (ZIC), were applied. Measurements were made for 188 μm × 141 μm areas with a resolution 0.046 μm in the Z axis. Several roughness (R) parameters were defined: Ra – arithmetical mean deviation, Rq – root mean square deviation, Rp – maximum profile peak height, Rv – maximum profile valley depth, Rsk – skewness, Rz – maximum height of profile, and Rku – kurtosis of profile. For each of these parameters the value minimum, maximum, mean, standard deviation and relative error has been determined.

### Gentamicin exclusion assay on Airway Surface Liquid infection model

NuLi-1 (Normal Lung, University of Iowa), derived from human airway epithelium of normal genotype, and a CF cell line, called CuFi-1 (Cystic Fibrosis, University of Iowa), derived from bronchial epithelium of a homozygous CFTR F508del/F508del individual, were kindly provided by Zabner (University of Iowa, Iowa City, IA). The ASL model was prepared according to methods described elsewhere by Zabner^31^. Both cell lines were inoculated with 25 μl of *P. aeruginosa* PAO1 reference strain (6.2 × 10^7^ cfu/ml), nonCF0038 isolate from burn wound (6.5 × 10^7^ cfu/ml) and CF708 small colony variant (1.0 × 10^6^ cfu/ml) at optical density at 600 nm of 0.1, and incubated for 1.5 h at 37 °C, 5% CO_2_. The 25 μl of KTN4 phage suspension (8.5 × 10^10^ pfu/ml) was added to each millicell hanging cell culture insert. Subsequently, cells were incubated for 1.5 h at 37 °C, 5% CO_2_. Next, cells were washed with PBS and apical washes were serially diluted in DMEM:F12 medium (Sigma-Aldrich) and quantified by viable counts on LB agar (Sigma-Aldrich) after 24 hours. To evaluate the ability of *P. aeruginosa* to invade into epithelial cells and the ability of phage KTN4 to prevent this invasion, extracellular and adherent bacteria were killed by addition of 400 μg/ml gentamicin (Thermo-Fisher Scientific) and incubated for 1 h at 37 °C, 5% CO_2_. The drug was subsequently removed by PBS washing and epithelial cells were lysed with 0.4% Triton-X100 (Sigma-Aldrich) 100 μl for 15 min at 37 °C, 5% CO_2_. The resulting lysate was serially diluted in DMEM:F12 medium and quantified by viable counts on LB agar after 24 hours. The gentamicin MICs for tested strains were as follows: 0.5 μg/ml, 2 μg/ml, and 64 μg/ml, for PAO1, nonCF0038 and CF708, respectively. Several controls of epithelial cells viability were prepared: (i) a negative control without any treatment, (ii) a negative control with TC media; (iii) a positive control with Triton-X100; (iv) 1.5 h after KTN4 treatment; (v) 1,5 h after strains treatment. The Nuli-1 and CuFi-1 cells were stained with 8 μM Calcien AM (live staining) (Life Technologies, NY, USA) and 3 μM propidium iodide (PI) (dead staining) (Life Technologies, NY, USA) according to manufacturer’s instructions. Negative controls (untreated) and positive controls (0.25% Triton-X100 treated) were included in the experimental set up. After staining, in all cases, filter inserts were XZ scanned using a confocal microscope (Zeiss LSM 510 Meta 40× objective, Jena, Germany). No toxicity influence of cell lines was noticed for phage and bacteria samples.

### Statistical analysis

The data were analyzed using the Statistica software package (StatSoft, Tulsa, OK, USA). All the values were expressed as mean ± SD and significant differences between variations (denoted p-values < 0.05) were found by means of the Snedecor-Fisher test using one-way ANOVA.

### *Galleria mellonella* larvae model

The *in vivo* assay was conducted on a wax moth larvae model according to the methodology described previously^18^. Briefly, larvae were inoculated with 10 μl lethal dose of bacterial cells established as follows: 10 CFU of PAO1 and nonCF0038 strains and 10^6^ CFU of CF708 isolate per larvae. After injection into the ventral side of the last pair of pseudopods, the larvae were incubated for 96/120 hours at 37 °C. For assessment of the antibacterial activity of KTN4 phage, larvae were sequentially injected with 10 μl of bacterial suspension and 10 μl of phage lysate at the titration equal to multiplicity of infection (MOI) 100. The results were read at 18, 24, 36, 48, 72 and 96/120 hours post injection and were expressed as the percentage survival rate assessed by macroscopic appearance. Experiments were performed in triplicate (10 larvae per trial). The controls consisted of uninfected and larvae receiving phage lysate only (negative) and infected with bacterial lethal dose (positive). The analysis of survival curves was performer by log-rank Mantel-Cox test. P-values < 0.05 were considered statistically significant. Statistical analysis was performed using GraphPad Prism software (GraphPad Software, Inc., La Jolla, USA).

## Additional Information

**How to cite this article**: Danis-Wlodarczyk, K. *et al*. A proposed integrated approach for the preclinical evaluation of phage therapy in *Pseudomonas infections*. *Sci. Rep.* **6**, 28115; doi: 10.1038/srep28115 (2016).

## Supplementary Material

Supplementary Information

## Figures and Tables

**Table 1:** Phage receptor identification on *P. aeruginosa* PAO1 mutants.

Bacterial strain	Phenotype	Origin	KTN4	φKZ
PAO1 (ATCC 15692)	Wild type	American Type Culture Collection	+	+
PAO1 Pirnay	Wild type with inactive type IV pili	Military Hospital Nederoverheembeek, Brussels, Belgium, Dr. Jean-Paul Pirnay	−	−
PAO1 Krylov	Wild type	Military Hospital Nederoverheembeek, Brussels, Belgium, Dr. Jean-Paul Pirnay	+	+
PAO1 Δrmd (A−, B+)	Deficiency in D-rhamnose biosynthesis; lack of A-band LPS	Laboratory of Foodborne Zoonoses, Guelph, Canada, Andrew M. Kropinski	+	+
PAO1 ΔrmLC (A−, B−, core-)	Deficiency in L-rhamnose biosynthesis; truncate core region, lack of A-band and B-band LPS	Laboratory of Foodborne Zoonoses, Guelph, Canada, Andrew M. Kropinski	+	+
PAO1 ΔwaaL (A−, B−)	Lack of WaaL ligating O-polymer to core-lipid A; LPS is devoid of A-band and B-band, semirough (SR-LPS, or core-plus-one O-antigen)	Laboratory of Foodborne Zoonoses, Guelph, Canada, Andrew M. Kropinski	+	+
PAO1 ΔwbpL (A−, B−)	Lack of glucosyltransferase WbpL essential for initiation of both A-band and B-band synthesis	Laboratory of Foodborne Zoonoses, Guelph, Canada, Andrew M. Kropinski	+	+
PAO1 ΔfliC ΔalgC ΔpilA	Lack of flagella; lack of AlgC required for A-band, core oligosaccharide, and alginate biosynthesis; lack of type IV pili	Technical University Hamburg, Germany, Max Schöbert	−	−
PAO1 ΔfliC wt algC ΔpilA	Lack of flagella; lack of type IV pili	Technical University Hamburg, Germany, Max Schöbert	−	−
PAO1 ΔfliC wt algC wt pilA	Lack of flagella	Technical University Hamburg, Germany, Max Schöbert	+	+
PAO1 wt fliC wt algC wt pilA	Wild type	Technical University Hamburg, Germany, Max Schöbert	+	+
