# Supplementary material for: A proposed integrated approach for the preclinical evaluation of phage therapy in Pseudomonas infections
Source: Sci Rep. 2016 Jun 15;6:28115. doi: 10.1038/srep28115 (PMC4908380; doi:10.1038/srep28115)
Supplement: Supplementary Information [file srep28115-s1.pdf]

A proposed integrated approach for the preclinical evaluation of phage therapy in *Pseudomonas* infections.

Katarzyna Danis-Wlodarczyk[1,2], Dieter Vandenheuvel[2], Ho Bin Jang[2], Yves Briers[2, 3], Tomasz Olszak[1], Michal Arabski[4], Slawomir Wasik[5], Marcin Drabik[6],Gerard Higgins[7,8], Jean Tyrrell[8], Brian J. Harvey[8], Jean -Paul Noben[9], Rob Lavigne[2*], Zuzanna Drulis-Kawa[1*]

Supporting information

Suppl. 1. Supplementary information of genome and proteome analysis of KTN4

Using high throughput sequencing by the Illumina MiSeq platform, the complete genome sequence was determined. KTN4 has a linear, circularly permutated and terminally redundant, A+T-rich (36,9% GC) double-stranded DNA molecule (279,593 bp). In total, 368 open reading frames (ORFs) could be predicted, varying in size from 36 to 2237 amino acid residues, as well as six tRNAs (Leu (UUA), Pro (UUG), Met (CAU), Asp (GUC), Asn (GUU), Thr (UGU) . Of these, 87 proteins have a predicted function. According to the orientation of transcription, ORFs are organized into operons and most are on a positive strand. The KTN4 shows a genome-wide nucleotide sequence similarity to: phiKZ 99%, PA7 99%, phiPA3 84%, 201phi2-1 78% (BLAST). As such, it can be defined as an isolate of the *Pseudomonas* phage phiKZ species. However, there are few significant differences. Genome of KTN4 lacks phiKZ gp24.1 (frame shift caused by two deletions) and gp24.2 (point mutation). In this position, there is a clear sequence of gp34 KTN4 on a positive strand. Also phiKZ gp70.1 is absent, showing only 58,27% nucleotide sequence similarity to the KTN4 genome. Two genes (KTN4 gp93 and gp94) show less than 60% nucleotide similarity to phiKZ genome and several genes have no similarity (KTN4 gp23-26, gp30-32, gp75, gp286-287, gp325, gp321). Finally, two additional proteins were annotated, which are also present in phiKZ genome and corrected according phiKZ RNA-seq (KTN4 gp14, 97.70% homology and gp59, 98,60% homology). None of these

genes have a predicted function. Based on phiKZ RNA-seq analysis performed previously by Ceyssens P.J·*et al.* (Ceyssens et al. 2014) and using the PISE EMBOSS fuzznuc program, 47 promoters were predicted for phage KTN4. Among them, 31 are early phage-specific promoters with highly conserved, uni-directionally distributed AT-rich intergenic motifs (5'-TATATTAC-3') (Fig. S2 A). Furthermore, less conservative upstream (5'-TTTaA-3') and downstream (5'-TG-3') motifs were found. The middle promoters are located on both strands and distributed throughout the whole genome. They are linked by only a weak AT-rich motif (5'-AAanntTAC-3'; lowercase letters represent a lower level of conservation) centered at position 24 with respect to the transcription start site (Fig. S2 B). For late transcription no sequence conservation upstream of 5' ends could be detected apart from a 5'-TATG-3' motif overlapping the transcription start site (11 late promoters) (Fig. S2 C). Using ARNOLD software, 107 of putative factor-independent terminators were predicted. Most potential stem-loop transcription terminators contain the tetranucleotide UUCG loops (Fig. S3). Using ESI-MS/MS analysis of proteins from denaturated phage particles fractionated on SDS-PAGE, 111 gene products have been identified, among which five virion-unrelated enzymes, 36 virion-associated proteins and 70 structural gene products, with sequence coverages ranging between 5,94% to 89% (Fig S4, Table S1). The KTN4 structural proteins were compared to their homologues from phiKZ (NC_004629.1 from 2008 and AF399011.1 from 2013), PA7 (JX233784.1), PhiPAK3 (HQ630627.1) and 201phi2-1 (NC_010821.1) phages (BLASTP). As expected, the highest similarity was found for phiKZ structural proteins, ranging from 63% to 100%, except for gp93, which was identical to PA7 hypothetical protein (AFO71119.1). Twenty two structural head proteins were identified including gp215 head protease identical to phiKZ gp175 and a major head protein gp155 with 98% similarity to phiKZ gp120 (Thomas et al. 2012), The contractile tail of phiKZ, the closes homologue to KTN4, is built from at least 32 different proteins, but a definitive structural function was assigned to only two of them: the

tail sheath protein (KTN4gp39 versus phiKZ gp29) and the tail tip protein (KTN4 gp166 versus phiKZ gp131). Tail tip protein is located at the periphery of the baseplate and possibly associates with fibers that emanate from the baseplate. In the ESI-MS/MS analysis two additional proteins were identified gp181 (putative tail sheath protein) and gp182 (putative tail fiber). The presence of tail associated enzyme gp221 (structural peptidoglycan hydrolase) corresponded to gp181 of phiKZ phage. This enzyme cleaves the host cell wall during the first stage of the life cycle (Briers et al. 2008). In the centre of the phiKZ baseplate, there is a density that resembles the needlelike ‘‘cell-puncturing’’ device of T4, which is most likely composed of gp181. Further analysis reveal gp52, that represents a chain A of monomeric subunit of Tubz, protein believed to be essential for the correct centering of replicated bacteriophage virions within the bacterial host. Moreover, four β/β′-like virion-associated proteins in KNT4 phage (gp106, gp185, gp219, and gp220) were assigned as a non-canonical multi-subunit viral RNA polymerase (RNAP) similar to phiKZ gp80, gp149, gp178, gp180 (Ceyssens et al. 2014). During ESI-MS/MS analysis we were not able to identify any peptide corresponding to gp202 (phiKZ gp164) and gp224 (phiKZ gp 184) (Ceyssens et al. 2014). Furthermore, the analysis of phiKZ RNAP performed by (Yakunina et al. 2015) allowed to identify five homologous subunits in KTN4 genome, the products of early phage genes. Four of these are cellular RNAP subunits homologs of the non-virion set (nvRNAP): 1) gp74, homologous to gp55 phiKZ, however shorter by 41 amino acids; 2) gp95 and gp96 (phiKZ gp71 and gp73); phiKZ gp72, a part of phiKZ subunit has no homologue in KTN4 genome; 3) gp97 identical to gp74 phiKZ and 4) gp158 identical to gp123 phiKZ. The fifth subunit, gp90 identical to gp68 phiKZ, is a protein of unknown function with no similarity to known RNAP subunits or any other known protein family. Gp74 and gp97 together correspond to msRNAP largest (bacterial β’) subunits and gp95 and gp96 subunit corresponds to the C-terminal half of msRNAP second largest (β in bacteria) subunits. Gp158 is highly diverged homolog of the N-terminal half of the second

largest (β in bacteria) msRNAP subunits. This complex initiates transcription from late promoters in rifampicin-resistant manner, tested *in vitro* in phiKZ, which suggests that virus relies on its own transcription machinery for the entire infection process. However, the late promoter 5'-TATG-3' conserved motif is necessary for transcription by nvRNAP *in vitro* (Yakunina et al. 2015). The endolysin of KTN4 (gp180) is highly identical (99%) to the endolysin of *P. aeruginosa* phiKZ gp144. This endolysin is well studied both at the molecular, biochemical (Briers et al. 2007; Briers et al. 2008; Cloutier et al. 2010) and structural level (Fokine et al. 2008). The consensus motifs for peptidoglycan binding (underlined) and the catalytic residue (boxed) are fully conserved in KTN4 gp180.

The protein sharing network for jumbo phage KTN4 is presented in Fig. 1. A resulting network comprises 495 phages (nodes) belonging to *Myoviridae*, *Siphoviridae*, *Podoviridae*, or uncharacterized and other phages and 6,948 relationships (edges) between them (Fig. 1A). In this graph, phage KTN4 was placed in a single component with five well-known *Pseudomonas* phiKZ-related phages including phiKZ, phiPA3, 201phi2-1, EL and OBP (Cornelissen et al. 2012), as well as phages phiJM-2012, SPN3US, CR5, and phiEaH2, which was separated from other components. When the network topology was computed with two classical measures such as the clustering coefficient (CL) and betweenness centrality (BC) (Brohée et al. 2008), this component shows the highest CL =1 (absolute cohesiveness) and the lowest BC = 0 (none of node acting as a bridge among other pairs of nodes). This network structure reflects the distinct core gene-sets shared between their genomes (Jang et al. 2013), which form a tight-knit clique of full interconnectivity. Next, the connectivity pattern of this component has been investigated not only on the basis of protein sequence identities, but also according to phage-phage similarity score after normalizing the number of shared genes between genomes (Leplae et al. 2010) (Lima-Mendez et al. 2008). As a result, over the threshold of 60% identity, phages KTN4, phiKZ, phiPA3, and 201phi2-1 kept forming an in-group relationship with phiKZ being the

closest relative to KTN4 (Fig. 1B). In addition, KTN4 was more closely related to phiKZ, phiPA3, and 201phi2-1 with the phage-phage similarity score ranging from 157.2 to 999.9, indicating more shared homologous genes between them than others. The other *Pseudomonas* phages including EL and OBP were connected for identity values less than 35% and similarity score, 79.9. The phages phiEaH2, SPN3US, CR5, and phiJM-2012, having different host ranges (i.e., *Erwinia*, *Salmonella*, *Cronobacter*, and *Vibrio* sp., respectively), were weakly connected to other phiKZ-related members in terms of both shared gene contents and sequence identity.

The genetic relationships of KTN4 have been investigated by constructing a mathematical model of gene (protein)-sharing network, extending to possible close relatives. In our phage population network, KTN4 is constricted to a single isolated component comprising the five *Pseudomonas* phiKZ-related phages and other potential relatives. Subsequent network decomposition strongly indicates that KTN4 belongs to the "phiKZ-like viruses", subdivision of the phiKZ-related groups (Cornelissen et al. 2012), with the large proportion of phiKZ-specific core gene-sets in common to the phiKZ, phiPA3, and 201phi2-1. More specifically, the connectivity patterns suggest that phiKZ appears to be the closest relative to KTN4 as their protein families with more than 90% sequence identity can be considered more recently shared than those of other phage members in this group (Halary et al. 2010). In addition, our population network can reveal other informative connections. The phages phiEaH2, SPN3US, and CR5 were found to interconnect solely with the phiKZ-related phages, indicating that they are probably diverged member of the phiKZ-related group as observed in phiJM-2012 (Jang et al. 2013). These results suggest that the phiKZ-like phages' diversity has not been fully delimited and that there are additional more distant relatives yet to be discovered.

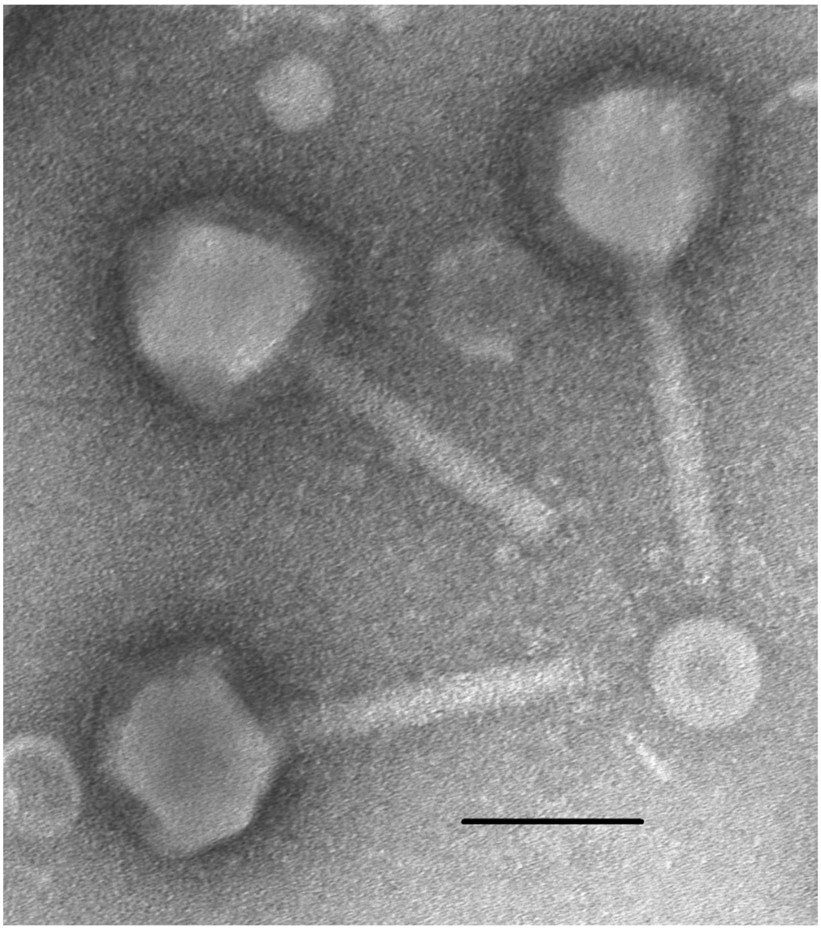

Fig. S1. Transmission electron microscopic images of phage KTN4. The scale bar represents 100nm.

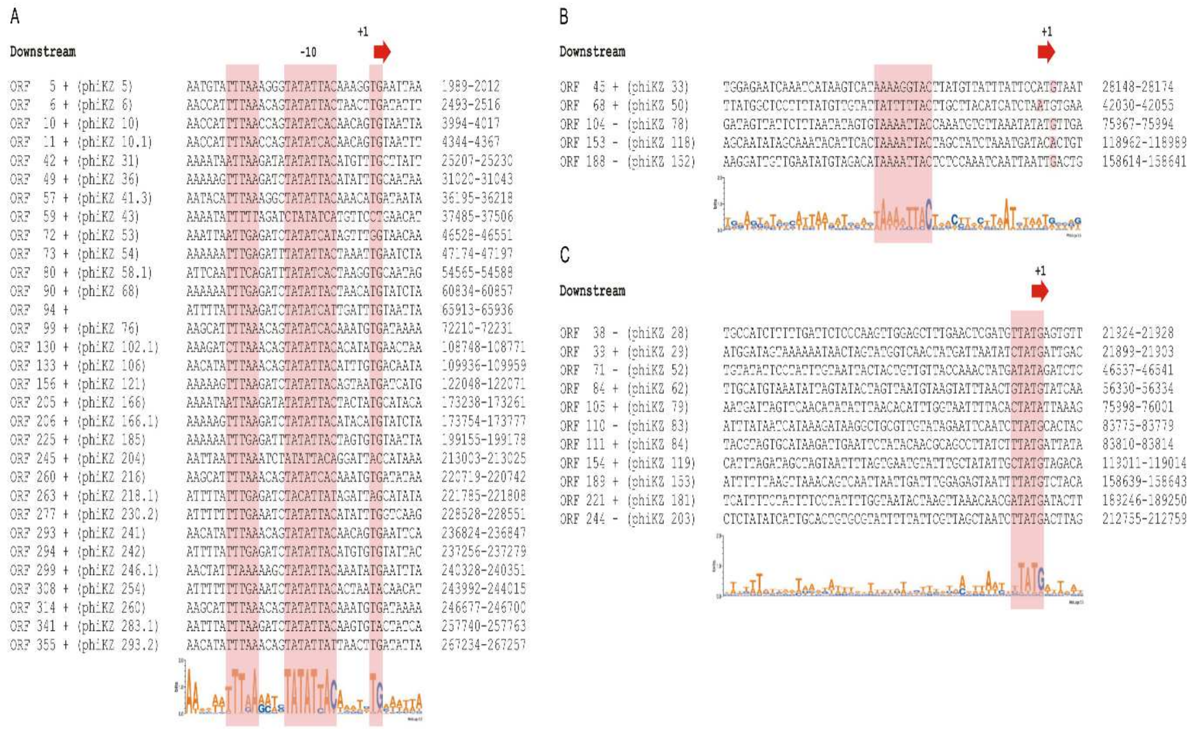

Fig S2. Alignments of KTN4 promoters. (A) Early promoters. The 5’ ends of primer extension products, which correspond to the transcription start sites, are located ~ 10 bp downstream from the center of the core TATATTAC motif and are associated with an additional conserved 5’-TG-3'motif. (B) Middle promoters. They are united by only a weak AT-rich motif (5’-AAanntTAC-3’; lowercase letters represent a lower level of conservation) centered at position 24 with respect to the transcription start site (C) Late promoters. No sequence conservation upstream of 5’ ends of late transcripts could be detected apart from a 5’-TATG-3’ motif overlapping the transcription start site. The corresponding sequence logos are depicted below the alignments. Pink bars delineate conserved promoter elements.

KTN4 Terminators

ORF	4	+	Erpin	+	ACATAATAATAGCCTTCCCCCAGGGGAAGGCtTTATGTCATGTAT	-17.40	1946-1965
ORF	8	+	Both	+	TGGCGACATACACCCTCCCTTCGGGGAGGGCTTTATTTTCAATA	-17.80	3569-3590
ORF	16	+	Both	+	CACGCAATAATGGGAGTCTTCGGACTCCCTTTTATTTTCTA	-16.10	7171-7188
ORF	18	+	Both	+	AACGATATAAATACCCTCCTTCGGGAGGGTATTTATTTTGTGA	-17.50	8763-8782
ORF	33	+	Rnamotif	+	GGCTATATGTCGTCAAGTTTAAGCTTGACTTTTTGCCCAAA	- 8.70	13835-13855
ORF	35	-	Both	-	TTAAATATAAAGCCCCCTAGGAACCACCTAGGGGGCTTTATAACGTCTT	-16.20	16500-16476
ORF	41	+	Both	+	TAAAGCATATCGCCTCCCTTCGGGGAGGCTTTATGTTGTTA	-18.50	25158-25175
ORF	43	+	Both	+	TAACAGCATAAGCCCTCCCATTGCGGGAGGGCaTTATGTTTATTT	-18.90	26845-26865
ORF	44	-	Both	-	ATAAACATAATGCCCTCCCGCAATGGGAGGGCTTATGCTGTTAAG	-17.80	26865-26845
ORF	46	+	Erpin	+	GTAAATATAATGCCCTCTCCATAAGGAGAGGGCTTATGATGTATTT	-17.70	29290-29311
ORF	48	+	Erpin	+	ATGAACATATTGCCTCCCCTAGGGGAGGCTTTATGCTGAGTT	-16.30	30981-30998
ORF	52	+	Both	+	TAACTTATAATGGAGCCCTTCGGGGCTCCTTTTATTTTTTT	-18.20	34542-34559
ORF	55	+	Both	+	TCGAAAGAGTCCACCCTGGATGGGGTGTTATATTAACTG	- 9.00	35551-35566
ORF	57	+	Both	+	TAATGGCATAAGCCCTCCCATAATGGGAGGGCaTTATGTCTTTTT	-19.20	36537-36557
ORF	58	-	Both	-	AAAGACATAATGCCCTCCCATTATGGGAGGGCTTATGCCATTAAG	-18.50	36557-36537
ORF	60	+	Erpin	+	TACTAAATAAAGCCCTCCTTATGGGGGGCTTTATACCGATTA	-14.70	39035-39054
ORF	67	+	Both	+	ATAACTATAAAGGAGCCATTATGGCTCCTTTTATGTTGTA	-11.80	42000-42016
ORF	68	+	Erpin	+	TAAGAAATATAGCCTCCCCTAGTGGGAGGCTTATATACTAAAA	-14.40	44397-44415
ORF	70	+	Both	+	ACGGCATATAACCCCCTCCAATTTAGGAGGGGGTTTATGTCACATT	-18.20	45385-45406
ORF	71	-	Erpin	-	TGTGACATAAACCCCCTCCTAAATTGGAGGGGGTTATATGCCGTTT	-18.70	45406-45385
ORF	73	+	Both	+	AAACTGATATAGCGGCTCCTTCGGGGGCCGCTTATATCTCCTT	-20.00	49476-49495
ORF	77	+	Both	+	AAATAAATTAATAGGGGAGTTAGACTCCCCTATTATTTCTTTGA	-14.50	53627-53646
ORF	81	+	Erpin	+	TTAAATAAATGAGAGTCCCGTACAGGGGCTCTcTTCTATCGTTTAG	-13.80	55484-55504
ORF	92	+	Both	+	CACGGCATAAACCAGGGGGCCTAAGGGTCCCCTGGcTTGCTTTAAAAC	-15.70	65499-65522
ORF	98	+	Both	+	GGCATAAATGATCCCTCCCTTAGTTGGGAGGGATTATGCTTTTTT	-17.10	72155-72176
ORF	101	+	Rnamotif	+	GGCGACATAAACCCCTCTCCATGTGGAGAGGGGTCTGTCTTTATT	-17.50	73283-73304
ORF	104	-	Rnamotif	-	AATAATGACGAGGTGGAAACTCCACCTTTCGTTCCTGA	- 8.50	75265-75251
ORF	107	+	Erpin	+	GTAAACATATTGCCTCCCCTAGGGGAGGCTTTATGCTGATTA	-16.30	79747-79764
ORF	109	+	Both	+	CACATCATAAAACCCTCCCATTACGGGAGGGTTTATTTCGTTAAC	-15.70	82201-82220
ORF	110	-	Both	-	TAACGAAATAAACCCTCCCGTAATGGGAGGGTTTTATGATGTGTC	-15.30	82220-82201
ORF	113	+	Both	+	TACAACATAATGCCCTCCCCTAGGGGAGGGCTTATGACCTTATT	-19.60	86840-86859
ORF	120	+	Both	+	ACAAACATAATGAGGAACCCTTCGGGGTTCCTCTTATGCTATGTAA	-15.30	96180-96200
ORF	122	+	Both	+	GACGACATATTGACGGGGCTCGGAAGAGTCCCGTCTTATGTTAGGAG	-14.90	99507-99530
ORF	124	+	Both	+	ACAAACATAACGGGGAGCCTTCATGGCTCCCTTTTATGCTGTTAG	-16.20	103225-103244
ORF	136	+	Both	+	TAACTGATAATGGGAGTCTTCGGACTCCCTTTTATTTTCTA	-16.10	111420-111437
ORF	142	+	Erpin	+	ATAGAAAAAAAACCACCCTATATAAGGGTGGTTTATAAAAGAGGT	-13.30	113888-113908
ORF	150	+	Rnamotif	+	TGAATCGGATCGCCCCGTAGGGGCTTTTATTTCATA	- 9.10	116426-116438
ORF	151_1	-	Rnamotif	-	AATCTACACTGTGTCTCCTTTTAGGGAGACAgTATGTATTAGCT	-12.10	116550-116531
ORF	151_2	-	Rnamotif	-	AAAGCATTTAAGCCCTCATAGGAGGGCTTTTATGTCGTA	-11.70	116704-116689
ORF	155	+	Both	+	AACAGACATAGGCCCTCCCTTCGGGGAGGGCTTTATGTTGTAT	-21.50	122002-122021
ORF	159	+	Both	+	TAAGCATGAAAGCCCCTCTTCGGAGGGGCTTATTTATGAGT	-18.40	127450-127467
ORF	160	-	Both	-	TAAAGACATAGCCCCTCTCCATGCGGAGAGGGGTTTATGCCATTAC	-17.20	128196-128175
ORF	164	+	Erpin	+	AACAACATAATGCCCTCCCATTACGGGAGGGCTTATGACGTTAAC	-18.20	134466-134486
ORF	165	-	Both	-	TAACGTCATAAGCCCTCCCGTAATGGGAGGGCaTTATGTTGTTTAG	-18.50	134486-134466
ORF	167	+	Both	+	ACCCGGCATAAGAGACACAGCCAAAAGCTGTGTCTCTTTTTGCTGTTT	-11.70	138555-138579
ORF	178	+	Both	+	AAAACTATAAAGCCTTCCTCTTCAAGAGGAAGGCTTTATAATGCTAT	-14.20	147015-147037
ORF	180	+	Rnamotif	+	TGTAACTTTTTGAGAGTCCTTCGGGACTCTCTTATGTTGTTAA	-17.30	148547-148566
ORF	182	+	Both	+	AACGACATATTGCCCTCCCTTCGGGGAGGGCTTTATTTTGTCT	-21.50	154331-154350
ORF	189	+	Both	+	TAACAGCATAAGCCCTCTCCCAAAGGAGAGGGCaTTATGTCGTACCA	-18.40	159606-159627
ORF	190	-	Erpin	-	TACGACATAATGCCCTCTCCTTTGGGAGAGGGCTTATGCTGTTATC	-17.70	159627-159606
ORF	194	+	Both	+	TGCATAAATGAGAGAGGGCAAAGGCCCTCTCTTATATTAATCT	-16.20	163817-163836
ORF	197	+	Both	+	TTTTAATTTAAAGCGGATCTGCTTTAAAGTGGATCCGCTaTTATTTCGTCTTA	-12.70	165695-165722
ORF	200	+	Both	+	TAACAGCATAAGCCCTCCCATAGTGGGAGGGCaTTATGTTCTTTA	-19.20	169233-169253
ORF	201	-	Both	-	AAGAACATAATGCCCTCCCACTATGGGAGGGCTTATGCTGTTAAA	-18.50	169264-169530
ORF	203	+	Both	+	AAGCAACATAAGCCCTCCCGCAATGGGAGGGCaTTATATTAGTAA	-18.50	172994-173014
ORF	204	-	Both	-	ACTAATATAATGCCCTCCCATTGCGGGAGGGCTTATGTTGCTTAT	-18.20	173014-172994
ORF	209	-	Erpin	-	AACGACATATAGCCCTCCCGTAATGGGAGGGCgTTATGCACTTAAT	-18.50	175215-175195
ORF	214	+	Both	+	TGACAGCATAAGCCCTCCCATTACGGGAGGGCaTTATGTCTTTTT	-18.90	179703-179723
ORF	215	-	Both	-	AAAGACATAATGCCCTCCCGTAATGGGAGGGCTTATGCTGTCATG	-17.80	179723-179703
ORF	218	-	Rnamotif	-	GGCACCACCAGCTCCCCATCTCTGAATGGGGAGTTTTTAACTGAG	-13.20	183101-183080
ORF	221	+	Both	+	AATCTATTAAAGGAGCCTCCCAATAGGGAGGTTCCTTATTTTGTCAA	-15.30	196004-196027
ORF	224	+	Both	+	TTGACATAAATGCCTCCCTTCGGGGAGGCTTTATTTCGTAT	-18.20	199116-199133
ORF	227	+	Both	+	CAATATAATAAGATCAGTGGGTGCATTGCCCACTGGTCaTTTTTTATATTAA	-12.00	200478-200504
ORF	230	+	Both	+	ACGATATAATAGAGTCCCCATTACGGGGACTCTTCTTTATAATT	-16.30	202265-202285
ORF	238	+	Both	+	GTTATATATTACCCTCTCCTTCGGGAGAGGGTTTTATGTTTAA	-19.20	206302-206321
ORF	239	+	Rnamotif	+	TACTTTTATAGGAGAGAACCATGATGGTTCTCTCTTTTTGACGTAA	-12.70	206931-206953
ORF	241	+	Both	+	AACAACATAATGCCCTCCCATTGTGGGAGGGCTTATGCCGTTAAA	-18.50	208132-208152
ORF	242	-	Both	-	TAACGGCATAAGCCCTCCCACAATGGGAGGGCaTTATGTTGTTTA	-19.20	208152-208132
ORF	245	-	Both	-	AATGGCATAAAGGAGCCCCTGGTGGGCTCCTTTAATGTAATAG	-14.40	212979-212959
ORF	247	+	Erpin	+	GATAACATATACCCTCCCCTTTATTGGGGAGGGaTTATTTACTTATA	-19.20	214043-214064
ORF	249	+	Erpin	+	AGCCAAAATAAACCCTCCTCTTAGTAGGAGGGTTTATATCGTCTCA	-13.20	215669-215690
ORF	261	+	Erpin	+	TGTTGTCATAAAGCCCTCCCTTCGGGGAGGGCTaTTGCACTAAATTT	-17.90	221747-221768
ORF	263	+	Both	+	TTTGAACAATAAGCGGTGTGTACACCGCTtTTATTCCGTGTTT	-11.70	222145-222162
ORF	265	+	Rnamotif	+	TTCGAGGCATATACTCCATGACTTATGTTGTGGGGTATATGTGTTATAA	- 8.50	223844-223869
ORF	267	+	Both	+	GTTTAATATTAAGGGACTCTTAATGAGAGTCCCTaTTTATTTTGCGTA	-14.10	224652-224673
ORF	269	+	Erpin	+	AAGACTGGGCAAAAGACATTCTAAAAGAGTGTTGGCTGCCCAGTCaTCTTTTCTGCCGT	-14.93	225691-225733
ORF	274	+	Both	+	GTAAAATAAATACCAGCCCTTCGGGGCTGGTTTATTTCATCTAG	-18.70	227846-227865
ORF	276	+	Both	+	AAGCATAAATAGGAGAGCTATTTAGCTCTCCTTTATTGCATTAA	-14.10	228489-228508
ORF	284	+	Both	+	TAACATAAAACGCCTCCCTTCGGGGAGGCTTTATTTTCCTT	-18.50	233224-233241
ORF	285	+	Both	+	AACAGTATATCGGGAGTCCATTTGGACTCCCTTTTATATCGTA	-16.70	234194-234213
ORF	291	+	Both	+	GATTGCATAAAGAGAGAGGATAAATTCCTCTCTCTTTTTATGTTGT	-14.40	236116-236138
ORF	292	+	Both	+	TACCATAAATACCTCCCTTCGGGGAGGTTTTATATTGTG	-14.70	236776-236791
ORF	293	+	Both	+	TAAGAGCATAAAGCCCTCCCTTCGGGGAGGGCTaTTGCACTAAATTT	-17.90	237218-237239
ORF	296	+	Both	+	TTGTATAATAAGAGCCCTTCGGGGCTCTTTCTATTTCGC	-15.10	239090-239105
ORF	298	+	Both	+	ACTTAATATACACCCTCCCTTCGGGGAGGGTtTTATTTATCTTTT	-19.00	240277-240296
ORF	306	+	Both	+	AGAAATAATTAGAGGACCTTCGGGTCCTCTTTTTAACCGAC	-16.30	243758-243775
ORF	307	+	Both	+	AAAGTCATAAACCCTCTCCCAAAGGAGAGGGcTTTTACATTAAA	-15.20	243954-243974
ORF	313	+	Both	+	GTTATTATATTGCCCTCCCATTGCGGGAGGGCTTATATTTTATT	-18.20	246626-246646
ORF	319	+	Both	+	ATTATGAAAATGGGGGTCTAAAGACCCCCTATCTATTTAAT	-15.10	249167-249184
ORF	322	+	Both	+	ACGCCATAATGGCCTCCCTTCGGGGAGGCTTTTATATCCCT	-18.20	250436-250453
ORF	329	+	Both	+	ATGCTTAATTAGGGAGGGTAATACCTCCCTTTTTTATATAA	-12.80	252726-252743
ORF	334	+	Both	+	ATGGTATAATTGCCCTCCTTCGGGAGGGCTTCTATTTCGTA	-18.20	254927-254944
ORF	342	+	Both	+	AATCTAATACATAAAGGGCTCTTCGGAGCCCTTTATTTTTTCTTTAT	-11.70	259065-259088
ORF	347	+	Both	+	CCAATGAGATACCCTCCCCTGATCGGGGAGGGTTTTTGACATCT	-17.90	263102-263122
ORF	348	+	Both	+	CTATTGATAATGCCCTCCCTTCGGGGAGGGCaTTATTTCGCTTAA	-22.00	263633-263652
ORF	351	+	Rnamotif	+	TACGGCATAAACCCCTCTCCGTATGGAGAGGGGaTATGTCTTTATT	-18.00	265810-265831
ORF	352	-	Both	-	TAAAGACATATCCCCTCTCCATACGGAGAGGGGTTTATGCCGTAAT	-17.10	265831-268510
ORF	355	+	Both	+	AAGATAACATAGAGCTAGCCCATTAGGGGCTAGCTCTTATATTTATTT	-14.70	267454-267478
ORF	356	-	Rnamotif	-	AATAAATATAAGAGCTAGCCCCTAATGGGCTAGCTCTATGTTATCTTA	-14.70	267478-267454
ORF	357	-	Both	-	AGCTAATTATAACCCTCCCATTATGGGAGGGTtTTTATCAGGTTAT	-16.00	268288-268268
ORF	358	-	Both	-	AATAAAATAAAAGCCCTCCATCACGGAGGGCTTTATGTTGTTATG	-15.70	268954-268934
ORF	359	-	Rnamotif	-	TGTGTCTGAATGCCAGGGGTTTGCCACCCCTGGCTATATTATGTCA	-18.00	269672-269650
ORF	360	-	Both	-	CATATTCTAAAGAGTAGCTTCGGCTACTCTTTTATGTTGCCA	-14.10	270872-270855
ORF	363	-	Both	-	CCATATACTAGCCTCCCACTTGGGGGAGGTTTTATACTGTC	-11.20	272004-271987
ORF	365	-	Both	-	GACGATATATCGCCTCCCTTCGGGGAGGCTTTATATTTTTTT	-18.50	273178-273161
ORF	366	-	Erpin	-	TAGACATAATGACCCTCCCTTAATTGGGAGGGTTTATGCTAACAAT	-16.50	275278-275257
ORF	368	-	Both	-	TTAAGTGTATACACCCTCCCTTAATTGGAGGGTGTTATTCCCAATTT	-17.10	276961-279249

Fig S3. Phage KTN4 predicted terminators with palindromes marked blue.

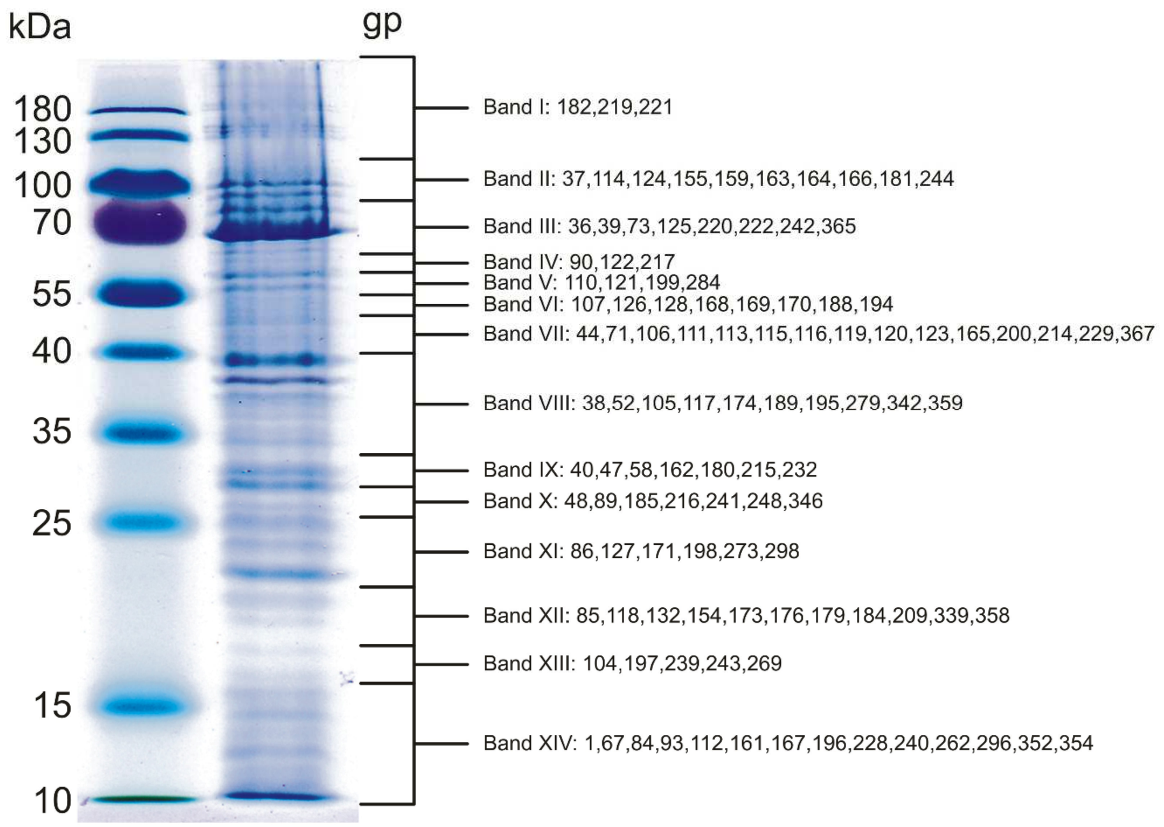

Fig. S4. SDS-PAGE pattern of KTN4 structural proteome against PageRuler Prestained Protein Ladder (Thermo Scientific) in first line. The corresponding molecular weight is mentioned left. The numbered fractions on the right, correspond to gel slices analyzed individually by ESI-MS/MS. The proteins are mentioned in the slice in which they were most abundantly present.

Table S1. ESI-MS/MS analysis of denaturated phage particles after fractionation on SDS-PAGE gel.

№	Protein	Identified function	Band №	Molecular weight (DA)		Number of identified peptides	Sequence coverage	Identity with other phages (BLASTP)			
				MS	SDS-PAGE			phiKZ	PhiPAK3	201phi2-1	PA7
1	gp1	Uncharacterized protein	14	14 673.80	14563.8	3	35.66%	99%	37%	39%	-
2	gp36	Structural head protein	3	62 440.10	62330.1	26	81.50%	99%	58%	54%	99%
3	gp37	Structural head protein	2	106 511.10	106401.1	32	55.70%	99%	57%	48%	-
4	gp38	Structural head protein	8	36 785.70	36675.7	13	63.44%	100%	49%	44%	99%
5	gp39	Tail sheath protein	3	79 511.20	79401.2	33	78.78%	99%	69%	63%	98%
6	gp40	Structural protein	9	32 658.70	32548.7	14	73.47%	100%	73%	76%	-
7	gp44	Structural head protein	7	50 478.80	50368.8	19	60.20%	98%	64%	54%	98%
8	gp47	Structural protein	9	30 253.60	30143.6	9	50.00%	99%	45%	40%	-
9	gp48	Structural head protein	10	29 956.50	29846.5	7	80.30%	98%	-	-	-
10	gp52	Chain A monomeric subunit of Tubz	8	36 567.20	36457.2	2	12.65%	99%	37%	31%	-
11	gp58	Uncharacterized protein	9	30 615.90	30505.9	6	32.44%	99%	71%	61%	-
12	gp67	Uncharacterized protein	14	15 500.60	15390.6	3	36.03%	98%	46%	44%	98%
13	gp71	Structural head protein	7	43 687.90	43577.9	13	51.92%	100%	55%	52%	99%
14	gp73	Uncharacterized protein	3	70 889.30	70779.3	14	33.01%	98%	48%	41%	98%
15	gp84	Uncharacterized protein	14	16 424.50	16314.5	6	71.32%	99%	37%	31%	-
16	gp85	Uncharacterized protein	12	20 854.40	20744.4	7	55.68%	95%	-	-	95%
17	gp86	Uncharacterized protein	11	24 057.30	23947.3	3	21.14%	96%	39%	32%	96%
18	gp89	Uncharacterized protein	10	28 962.10	28852.1	9	47.56%	100%	55%	53%	-
19	gp90	Non-virion DNA-dependent RNA polymerase subunit*	5	60 527.30	60417.3	2	7.90%	99%	58%	50%	99%
20	gp93	Uncharacterized protein	14	14 936.80	14826.8	5	70.83%	-	-	36%	100%
21	gp104	Uncharacterized protein	13	19 031.70	18921.7	4	33.13%	96%	-	26%	98%
22	gp105	Structural head protein	8	34 798.90	34688.9	12	63.60%	99%	47%	38%	99%
23	gp106	β/β′-like virion-associated proteins	7	50 834.20	50724.2	16	55.95%	99%	65%	56%	99%
24	gp107	Structural protein	6	54 795.30	54685.3	23	80.47%	99%	37%	31%	99%

25	gp110	Structural head protein	5	56 385.00	56275	12	42.68%	97%	85%	-	98%
26	gp111	Structural head protein	7	47 327.00	47217	19	64.16%	100%	37%	34%	-
27	gp112	Structural head protein	14	17 092.70	16982.7	7	83.89%	99%	35%	33%	-
28	gp113	Structural head protein	7	49 280.00	49170	15	43.06%	99%	25%	26%	99%
29	gp114	Structural protein	2	110 487.40	110377.4	30	50.57%	99%	58%	52%	99%
30	gp115	Structural protein	7	40 628.10	40518.1	7	28.53%	99%	58%	56%	-
31	gp116	Structural head protein	7	44 414.80	44304.8	14	51.71%	98%	63%	54%	-
32	gp117	Structural head protein	8	36 014.60	35904.6	19	78.96%	100%	49%	47%	-
33	gp118	Structural head protein	12	21 586.50	21476.5	6	52.27%	99%	53%	51%	-
34	gp119	Structural head protein	7	50 842.60	50732.6	17	50.00%	99%	38%	35%	99%
35	gp120	Structural head protein	7	48 412.40	48302.4	16	44.03%	98%	26%	-	98%
36	gp121	Structural head protein	6	56 940.00	56830	16	51.51%	98%	27%	30%	97%
37	gp122	Structural head protein	4	59 192.20	59082.2	26	64.11%	98%	-	-	97%
38	gp123	Structural head protein	7	42 552.20	42442.2	11	35.70%	99%	34%	28%	-
39	gp124	Structural head protein	2	89 012.60	88902.6	20	39.43%	97%	31%	29%	99%
40	gp125	Uncharacterized protein	3	63 780.40	63670.4	6	13.76%	99%	48%	42%	-
41	gp126	Structural head protein	4	55 292.40	55182.4	12	37.53%	99%	59%	54%	-
42	gp127	Uncharacterized protein	11	22 993.10	22883.1	3	25.93%	97%	47%	36%	-
43	gp128	Structural protein	4	52 562.30	52452.3	7	22.81%	99%	44%	42%	-
44	gp132	Uncharacterized protein	12	20 422.20	20312.2	3	29.01%	99%	64%	66%	-
45	gp154	Structural head protein	12	21 118.50	21008.5	8	67.80%	100%	52%	53%	-
46	gp155	Major head protein	2	83 044.70	82934.7	27	72.03%	98%	67%	63%	98%
47	gp159	Uncharacterized protein	2	81 323.90	81213.9	7	12.80%	98%	43%	34%	98%
48	gp161	Structural protein	14	16 948.80	16838.8	4	43.45%	96%	-	35%	99%
49	gp162	Structural protein	9	33 581.10	33471.1	10	38.19%	98%	40%	36%	99%
50	gp163	Structural head protein	2	84 447.10	84337.1	19	38.23%	99%	57%	49%	99%
51	gp164	Structural protein	2	100 450.10	100340.1	19	33.75%	97%	56%	55%	-
52	gp165	Structural protein	7	48 629.30	48519.3	11	37.94%	99%	53%	54%	-
53	gp166	Tail tip protein	2	85 168.40	85058.4	24	51.53%	97%	41%	38%	-

54	gp167	Structural protein	14	12 741.90	12631.9	5	54.72%	95%	46%	45%	-
55	gp168	Structural protein	6	53 074.90	52964.9	11	32.97%	99%	40%	38%	99%
56	gp169	Structural protein	6	51 629.20	51519.2	10	35.23%	99%	39%	35%	28%
57	gp170	Structural protein	6	52 794.50	52684.5	17	54.49%	98%	47%	38%	25%
58	gp171	Uncharacterized protein	11	24 417.30	24307.3	6	42%	100%	36%	35%	-
59	gp173	Uncharacterized protein	13	20 064.30	19954.3	3	22.22%	99%	46%	40%	-
60	gp174	Structural protein	8	33 824.80	33714.8	12	73.65%	99%	70%	66%	-
61	gp176	Structural protein	12	19 664.70	19554.7	2	12.80%	98%	30%	-	-
62	gp179	Structural protein	12	20 550.00	20440	7	43.43%	98%	45%	45%	99%
63	gp180	Endolysin*	9	30 282.90	30172.9	8	46.03%	97%	47%	44%	51%
64	gp181	Putative tail sheath protein	2	85 190.00	85080	31	77.35%	98%	55%	41%	98%
65	gp182	Putative tail fiber protein	1	116 150.10	119130	38	72.58%	97%	36%	28%	97%
66	gp184	Uncharacterized protein	12	19 939.70	18480	4	30.36%	98%	25%	-	-
67	gp185	β/β′-like virion-associated protein	10	26 098.10	24640	11	63.84%	99%	67%	62%	-
68	gp188	Uncharacterized protein	6	55 385.50	52470	5	14.46%	99%	80%	70%	-
69	gp189	Structural head protein	8	34 215.70	33000	14	89%	99%	32%	28%	-
70	gp194	Structural head protein	6	51 221.40	48510	25	86.17%	99%	58%	53%	-
71	gp195	Uncharacterized protein	8	36 016.30	34760	9	39.87%	100%	39%	38%	-
72	gp196	Uncharacterized protein	14	16 784.10	14740	3	52.24%	99%	26%	-	99%
73	gp197	Structural protein	13	17 896.10	17270	6	65.60%	99%	-	-	-
74	gp198	Uncharacterized protein	10	24 279.80	22990	8	66.99%	99%	46%	42%	-
75	gp199	Structural head protein	5	58 593.30	56980	22	51.93%	99%	-	-	-
76	gp200	Structural head protein	7	44 512.30	43010	16	60.10%	99%	52%	44%	-
77	gp209	Uncharacterized protein	10	20 225.00	18590	3	22.48%	99%	45%	35%	-
78	gp214	Structural protein	7	41 360.80	38720	13	73.01%	99%	41%	38%	99%
79	gp215	Head protease	9	31 928.10	29700	12	64.07%	100%	60%	56%	-
80	gp216	Structural head protein	10	27 850.00	26400	9	60.42%	100%	70%	63%	100%
81	gp217	Structural head protein	5	61 170.50	56210	19	72.99%	98%	48%	41%	-
82	gp219	β/β′-like virion-associated protein	1	167 516.50	158510	51	51.56%	99%	58%	53%	-

83	gp220	β/β′-like virion-associated protein	3	63 084.20	59840	18	49.63 %	97 %	75%	68%	98 %
84	gp221	Structural peptidoglycan hydrolase	1	246 430.90	242330	62	37.90 %	98 %	37%	34%	98 %
85	gp222	Structural protein	3	77 193.00	71720	17	37.42 %	97 %	64%	63%	98 %
86	gp228	Uncharacterized protein	14	13 039.60	11990	2	24.22 %	100 %	-	-	-
87	gp229	Thymidylate kinase*	7	40 994.40	38720	12	49.15 %	100 %	35%	32%	-
88	gp232	Uncharacterized protein	10	30 790.70	28710	3	18.77 %	97 %	-	-	95 %
89	gp239	Uncharacterized protein	13	19 305.20	17600	3	23.75 %	98 %	46%	41%	-
90	gp240	Structural protein	14	17 449.40	15730	2	19.58 %	100 %	60%	62%	-
91	gp241	Uncharacterized protein	10	25 888.40	24090	6	38.36 %	99 %	43%	36%	98 %
92	gp242	Structural protein	3	72 744.80	70950	13	28.68 %	99 %	34%	28%	98 %
93	gp243	Structural protein	13	18 071.50	17160	4	57.05 %	100 %	-	-	99 %
94	gp244	Structural protein	2	83 120.40	76670	23	37.45 %	99 %	58%	51%	99 %
95	gp248	Uncharacterized protein	10	29 676.40	25960	2	10.17 %	100 %	-	24%	-
96	gp262	Uncharacterized protein	14	10 000.80	7590	2	24.64 %	97 %	-	-	97 %
97	gp269	Structural protein	14	17 955.30	15070	4	35.04 %	100 %	41%	33%	99 %
98	gp273	Structural protein	8.9	23 729.20	21890	4	20.60 %	100 %	-	-	-
99	gp279	Uncharacterized protein	8	35 239.80	33220	2	14.24 %	100 %	-	-	-
100	gp284	Thymidylate synthase*	5	58 214.90	53680	2	5.94 %	99 %	46%	60%	-
101	gp296	Structural protein	14	15 184.00	12980	4	50.85 %	99 %	32%	-	98 %
102	gp298	Uncharacterized protein	11	25 232.70	23100	2	15.24 %	96 %	-	-	-
103	gp339	Uncharacterized protein	12	20 495.30	18810	3	19.30 %	100 %	57%	57%	-
104	gp342	Uncharacterized protein	8	34 070.20	31900	5	19.65 %	93 %	-	-	97 %
105	gp346	Uncharacterized protein	12	26 364.50	23870	2	11.06 %	98 %	48%	40%	99 %
106	gp352	Uncharacterized protein	14	17 830.30	14190	8	66.67 %	88 %	-	-	97 %
107	gp354	Uncharacterized protein	14	7 495.30	6380	2	32.76 %	91 %	-	-	95 %
108	gp358	Uncharacterized protein	12	21 849.80	19140	2	16.09 %	63 %	60%	30%	63 %
109	gp359	Structural protein	8	37 136.40	36630	8	47.75 %	97 %	53%	-	96 %
110	gp365	Structural protein	3	72 814.60	68090	19	45.23 %	76 %	-	-	98 %
111	gp367	Ribonucleoside reductase*	7	45 827.80	41910	9	28.08 %	98 %	75%	64%	-

*enzymes non-associated with phage particles

Table S2. Phage activity comparison of fourteen different *Pseudomonas* phages on *P. aeruginosa* strains from Military Hospital Nederoverheembeek, Brussels, Belgium collection [Pirnay JP et al., 2002].

	***PA* strains**	**KTN4**	**KTN6**	**KT28**	**KMV**	**LKD16**	**LUZ19**	**LKA1**	**LIT1**	**LUZ7**	**LUZ24**	**LSL4**	**LMA2**	**LBL3**
1	US449	■					■			■			■	
2	LMG14083	■	■	■										
3	Bu007	■	■	■								■		■
4	PAO23		■	■		■	■			■	■			■
5	Aa 249													
6	US448	■	■	■			■		■		■	■	■	■
7	PAO1 Krylov	■	■	■	■	■	■	■		■	■			■
8	Lo050									■	■			■
9	US450		■	■	■	■	■			■	■			■
10	Li004	■	■		■	■	■		■					
11	Be128		■	■	■					■	■	■		
12	Lo053		■	■										
13	ATCC 27853	■	■	■	■	■	■		■	■				■
14	Br906	■	■	■	■				■	■		■	■	■
15	PhDW6	■	■	■	■	■	■		■	■			■	■
16	So099		■	■							■			
17	Aa 245		■	■	■								■	
18	LMG5031									■				■
19	C17	■	■		■	■	■							
20	C19			■						■				
21	Lo049													
22	Br642	■	■	■								■	■	■
23	Li012			■								■		
24	Is579		■	■										
25	C	■	■	■						■				
27	Lw1047								■					
28	Br257	■	■		■	■	■		■	■	■			■
29	Br667													
30	C18	■	■		■	■	■							
31	LMG2107	■	■	■	■	■	■			■	■	■	■	■
32	Bu004													
33	C1	■	■	■			■			■				
34	PAO29													
35	C13		■	■							■			■
36	C2	■												■
37	Aa 246		■	■	■	■	■			■			■	■
38	LMG14084	■	■	■										
39	Br735			■										
40	Mi162		■	■						■				■
41	SG17M	■	■		■	■	■				■			■
42	Pr335	■	■	■	■	■	■						■	■
43	Is580	■	■	■								■	■	■
44	Li009	■	■							■			■	
45	Be136	■	■	■	■	■	■	■		■		■	■	■
46	Is573		■											■
47	Br908		■										■	
48	TuD199		■	■	■	■	■			■				■
49	So095			■										
50	So092			■								■		■
51	Br229					■	■							
52	SG50M	■	■	■										■
53	PT31M	■	■		■	■	■				■			■

54	Mi151													
55	US447													
56	Bo548													
57	Br680													
58	PA6													
	Summary	27	39	34	19	18	23	2	7	20	13	10	14	27
	% of the tested strains	46.6	67.2	58.6	32.7	31.0	39.7	3.5	12.1	34.5	22.4	17.2	24.1	46.6

grey box –active, white box – no activity.
